# Advanced superimposition method to evaluate the marginal and internal fit of ceramic crowns fabricated using heat pressing techniques

**DOI:** 10.7717/peerj.19117

**Published:** 2025-04-03

**Authors:** Nasser M. Alqahtani, Saurabh Chaturvedi, Mohamed Khaled Addas, Manar Fahad A. AlQahtani, Arwa Ahmed M. Alhudiry, Shahrah H. Qahtani, Mohammad A. Zarbah, Asim Elsir Elmahdi, Marco Cicciù, Giuseppe Minervini

**Affiliations:** 1 Department of Prosthetic Dentistry, College of Dentistry, King Khalid University, Abha, Aseer, Saudi Arabia; 2 Department of Biomedical and Surgical and Biomedical Sciences, Catania University, Catania, Italy; 3 Saveetha Dental College and Hospitals, Saveetha Institute of Medical and Technical Sciences (SIMATS), Saveetha University, Chennai, Tamil Nadu, India; 4 Multidisciplinary Department of Medical-Surgical and Dental Specialties, University of Campania Luigi Vanvitelli, Caserta, Italy

**Keywords:** Digital dentistry, CAD-CAM, 3D Printing, Intra oral scan, All-ceramic crown, Permanent crown, Dental software

## Abstract

**Background:**

Digital technologies can enhance the success of permanent crowns. The present study aimed to evaluate marginal adaptation and internal fit of permanent crowns fabricated using stereolithography (SLA) and digital light processing (DLP) 3D printing technologies following scans using two different intraoral scanners (IOS) (Medit i700, shape (TRIOS 3)).

**Methods:**

Three typodont #14 teeth were prepared for full veneer all ceramic crowns with three types of margins—chamfer (CFL), rounded shoulder (RSFL) and rounded shoulder with bevel (RSBFL). A total of 360 study models were made and divided into two groups based on the type of intraoral scanner used for digital impressions. Group A in which the intraoral scanner MEDIT i700 was used, and Group B in which intraoral scanner TRIOS 3 was used (n = 360/group). The subgroups were made as Medit i700-SLA, Medit i700-DLP, TRIOS 3-SLA and TRIOS 3-DLP (n = 180/subgroup). These were further divided into three sub-subgroups based on the finish lines: CFL, RSFL, and RSBFL (n = 30 each sub-subgroups). All ceramic crowns were made on models and scanned to assess discrepancies (marginal adaptation and internal fit) at nine zones (Z1–Z9). Three-way analysis of variance and pairwise comparison was done (Tukey HSD test) (*α* = 0.05).

**Results:**

The mean marginal gap and internal fit values were lowest for Group A-1 with RSBFL in each zone. The intergroup comparison showed values for marginal gap and the internal fit were minimum for Group A-1 for each finish line design, with the lowest for RSBFL at zone Z2 0.04 ±0.001.

**Conclusions:**

Regardless of the IOS and 3D printing techniques, the smallest gap was observed in RSBFL, followed by RSFL and CFL. The all-ceramic crowns fabricated using a Medit intraoral scanner with an SLA 3DPrinter with a rounded shoulder finish line had the best marginal and internal fit.

## Introduction

Patient expectations have evolved, with individuals now seeking a deeper understanding of procedures and presenting clearer and more detailed requests for specific outcomes to their dentists. As a result, dentists are finding it necessary to personalise each patient’s experience (P et al., 2024). With the help of digital tools, dentists can customise every aspect of treatment, from planning to using visual aids to communicate with patients through to the procedure itself (Alqahtani et al., 2024; Patil et al., 2023). A permanent all ceramic crown is one a common treatment modality.

The survival of a permanent crown is predominantly influenced by marginal integrity and retention (Roy et al., 2021; Al Wadei et al., 2022). Manual and laboratory variables play a significant role, and errors are mainly influenced by laboratory factors (Al Wadei et al., 2022; Chaturvedi et al., 2021b; Alsubaiy et al., 2021; Chaturvedi et al., 2020). These errors in permanent crowns can be reduced or eliminated with the use of advanced technologies in digital dentistry like digital impressions, CAD-CAM, and 3D printing.

The fit of the permanent crown relies on the method of preparation, the materials used and the morphology of prepared teeth. All ceramic crown is a type of permanent crown that ensures high esthetics. Its success depends on various factors, along with the proper internal adaptation and marginal fit (Elrashid et al., 2019). Various all-ceramic systems have been proposed for the fabrication of all ceramic crowns: aluminous core ceramics, slip-cast ceramics, heat-pressed ceramics and machined ceramics (Chaturvedi et al., 2021b). Heat-pressed ceramic is one of the commonly used ceramic systems. It utilises the lost wax technique for the fabrication of all ceramic crowns. In this, the wax pattern is fabricated manually over the master model of the prepared tooth (Chaturvedi et al., 2021b; Chaturvedi et al., 2020; Elrashid et al., 2019; Alqahtani, 2017). During this procedure, the chances of error incorporation are very high because these techniques are done manually, there is dependence on the technician’s manual skills and various steps of wax and other material manipulation, which would ultimately affect the fitting of all ceramic crowns and their success.

These errors in the fabrication of wax patterns can be overcome with advancements in dentistry, such as the use of the milling/subtractive method or 3 Dimensional (3D) printing/additive method, for the fabrication of patterns for investment (Chaturvedi et al., 2021a). The problem with the subtractive method is the wastage of material, the inherent restrictions related to the machining tools, and the properties of the materials used. In 3D printing technology, for each millimeter of material, there are 5–20 layers, which the machine lays down as successive layers—to build a designed shape by segmenting the 3D model data (CAD design) into multi-slice images (Chaturvedi et al., 2020; Van Noort, 2012; Abduo, Lyons & Bennamoun, 2014). Additionally, compared to milling, 3D printing uses a lot less material, there is virtually no material loss, and leftover material can be used in the future (Van Noort, 2012). Also, the advanced 3D printing compatible castable resin can be printed to make patterns for all-ceramic crowns at a time with fine-detail reproduction (Abduo, Lyons & Bennamoun, 2014).

For using advanced digital techniques like 3D printing, initially, the virtual model of the prepared tooth is required. This can be achieved by using intraoral scanners (IOS) (Alqahtani et al., 2024; Al-Imam et al., 2019). These intraoral scanners make the digital impression of the prepared tooth and store it in DICOM file format, which can be converted into standard tessellation language (STL) file format to use in CAD procedure and later in the 3D printing software. The intraoral scanner reduces the operators’ errors linked with impression material handling and/or recording of impressions, as most of the work is done by the scanner itself (Alqahtani et al., 2024), while the 3D printing technique helps in reducing the laboratory errors affecting the fit of the permanent crown. Intraoral scanners and 3D printing technologies are currently used for the fabrication of fixed/removable dental prostheses, surgical guides for implants, orthodontic models and surgical planning in maxillofacial area and even in recording the centric relations. Various commercial intra-oral scanners are available and vary in terms of accuracy (Kwon et al., 2021; Suese, 2020). Similarly, various types of 3D printing are available like fused deposition modelling (FDM), stereolithography (SLA/SLG), selective laser sintering (SLS), selective laser melting (SLM), powder binder printers (PBP), and digital light processing (DLP) but the most used in dentistry are SLA and DLP type (Anadioti, Kane & Soulas, 2018; Rezaie et al., 2023). In the DLP process, a light source from a projector is allowed to cure the liquid resin in a layers-by-layer fashion, creating a designed object layer-wise in an upside-down manner. On the other hand, the SLA 3D printer uses ultraviolet lasers to polymerise photosensitive resin. To create the 3D structure, these photopolymers are exposed to a UV laser beam directly while the stage moves in different directions (Rezaie et al., 2023).

Any permanent crown’s success depends heavily on internal adaptation and marginal fit (Peng, Chung & Ramos, 2020; Rahmé et al., 2008; Peng et al., 2020). A good internal adaptation enhances the retention form, resistance form, and durability of the permanent crown (Peng, Chung & Ramos, 2020; Sadeqi, Baig & Al-Shammari, 2021; Martins et al., 2012), while a good marginal fit reduces microleakage, prevents cement breakdown, minimises plaque formation, and limits secondary caries (Chaturvedi et al., 2020; Peng et al., 2020). The finish line geometry affects the marginal and internal fit of the crown. The commonly used finish lines are chamfer, rounded shoulder, and rounded shoulder with bevel designs. Each design has distinct characteristics that impact crown fit, marginal adaptation, and overall durability. The chamfer finish line has its importance in the sense of its conservative preparation style, preserving more tooth structure compared to other designs. This margin type provides adequate material bulk while minimising stress concentration at the crown’s edge. Chamfer margins have been shown to allow good marginal adaptation and compatibility with a variety of materials, particularly ceramics (Pjetursson et al., 2007; Edelhoff & Sorensen, 2002). The rounded shoulder margin offers greater thickness at the marginal edge, beneficial for ceramic restorations requiring additional support, reducing the risk of chipping along the margin. Literature supports the rounded shoulder as an effective design for enhancing fit and stability in ceramic restorations, ideal for balancing strength and esthetics (Shillingburg, 2012; Magne & Douglas, 1999). A rounded shoulder with a bevel margin combines the advantages of a shoulder margin with an added bevel, enhancing the marginal seal and allowing the cement to flow more effectively, reducing the gap at the crown edge. This design compensates for minor preparation inaccuracies and provides good adaptability and a strong marginal seal, particularly for crowns subject to high-stress areas (Rosenstiel, 2006).

To examine these two factors in temporary crowns, numerous research studies have been done with varying degrees of success (Chaturvedi et al., 2020; Peng, Chung & Ramos, 2020; Peng et al., 2020; Sadeqi, Baig & Al-Shammari, 2021; Sidhom et al., 2022; Sampaio et al., 2021; Mohajeri et al., 2021). Different methods have been employed to measure the marginal adaptation and internal fit in previous research: the direct-view method (Holden et al., 2009), cross-sectional method (Sachs et al., 2014), silicone replica technique (Ryu et al., 2020), triple scan method/superimposition method (Holst et al., 2011), micro-computed tomography (Dauti et al., 2019), optical coherence tomography (Al-Imam et al., 2019). Each of these methods has its advantages and drawbacks. Direct-view methods use microscopic technologies and involve human error; the cross-sectional method results in the destruction of samples, micro-computed tomography requires an X-ray source and dedicated reconstruction software similarly, optical coherence tomography uses non-invasive optical scattering technology emitting coherent light to measure multiple points with micrometric resolution, although beneficial but expensive and availability is challenging (Ayres et al., 2024). On the other hand, the superimposition method is more advanced compared to direct-view, cross-sectional and silicone replica methods. It is easily available in most clinics and laboratories and is comparatively more cost-effective than micro-computed and optical coherence tomography. It involves the method of scanning the internal and external aspects of the prosthesis and the abutment tooth, respectively, to obtain 3D data and measure the marginal and internal fit by overlapping the 3D data on an analysis software. It is a non-destructive, non-radioactive method capable of providing reproducible results at any time by scanning the data (Ayres et al., 2024; Son et al., 2019). This advanced superimposition method was utilised in the present study to assess the marginal adaptation and internal fit. In a recent study, Lee, Lee & Lee (2017) examined the internal fit of full veneer interim crowns fabricated with CAD/CAM milling and 3D printing systems. Results showed that mean discrepancy values in three groups were 171.6 (97.4) µm for the crown manufactured by the milling system and 149.1 (65.9) and 91.1 (36.4) µm, respectively, for the crowns manufactured with the two types of 3D printing system. It was concluded that the internal and marginal fit of the crowns produced by 3D printing systems was much better than that of the crowns produced by CAD/CAM milling systems (Lee, Lee & Lee, 2017). None of the studies have compared permanent crowns’ marginal adaptation and internal fit, especially when fabricated with advanced technologies.

Thus, the present study was conducted to evaluate the marginal adaptation and internal fit of the permanent crown (pressable ceramic crowns) fabricated using SLA and DLP technologies after scanning with two different intraoral scanners (Medit i700 (Medit Link v2.4.4, Medit, Seoul, Korea, 2021) and three shapes (TRIOS 3)) using superimposition technique. The null hypothesis assumed that the fit of permanent crown fabricated using different 3D printing techniques and IOS would not differ.

## Materials & Methods

### Study design

**Figure 1:**
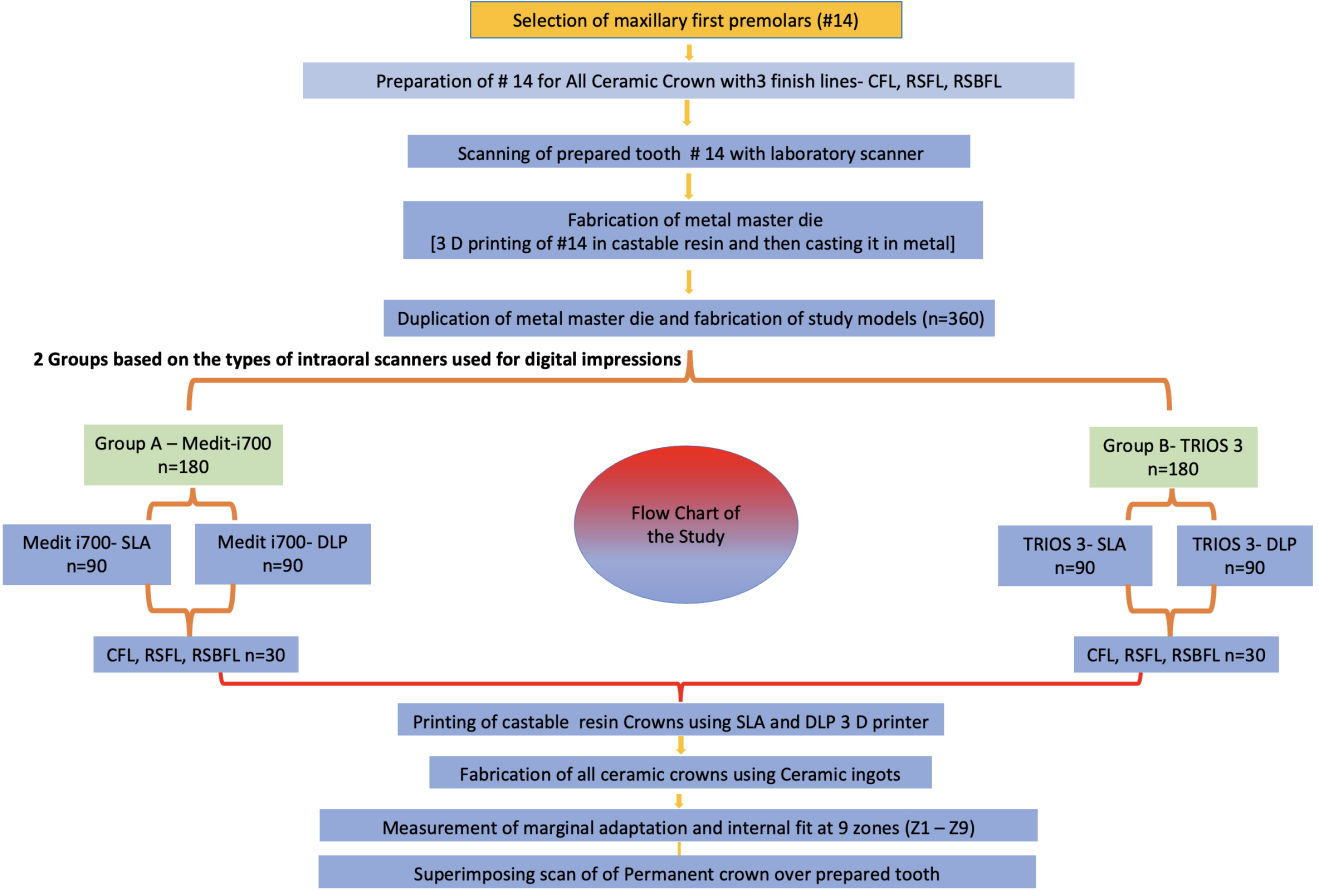
Flow chart of the study.

The study was a pure *in-vitro* study and designed to evaluate the marginal adaptation and internal fit of a premolar permanent all ceramic crown fabricated using advanced techniques. It was conducted at the Department of Prosthetic Dentistry, College of Dentistry, King Khalid University and the ethical waiver was obtained from the institute committee (IRB/KKUCOD/ETH-W/2022-23/005). For conducting the study, a flow chart was made and followed meticulously (Fig. 1).

Three typodont pre-molar teeth of the maxillary arch (#14) were fixed in three different typodont jaw with their adjacent teeth (#15, #13). The #14 preparation was done for full veneer all ceramic crowns with three types of margins—chamfer (CFL), rounded shoulder (RSFL), and rounded shoulder with bevel (RSBFL). The occlusal preparation of 1.5 mm and taper of six degrees was done.

**Figure 2:**
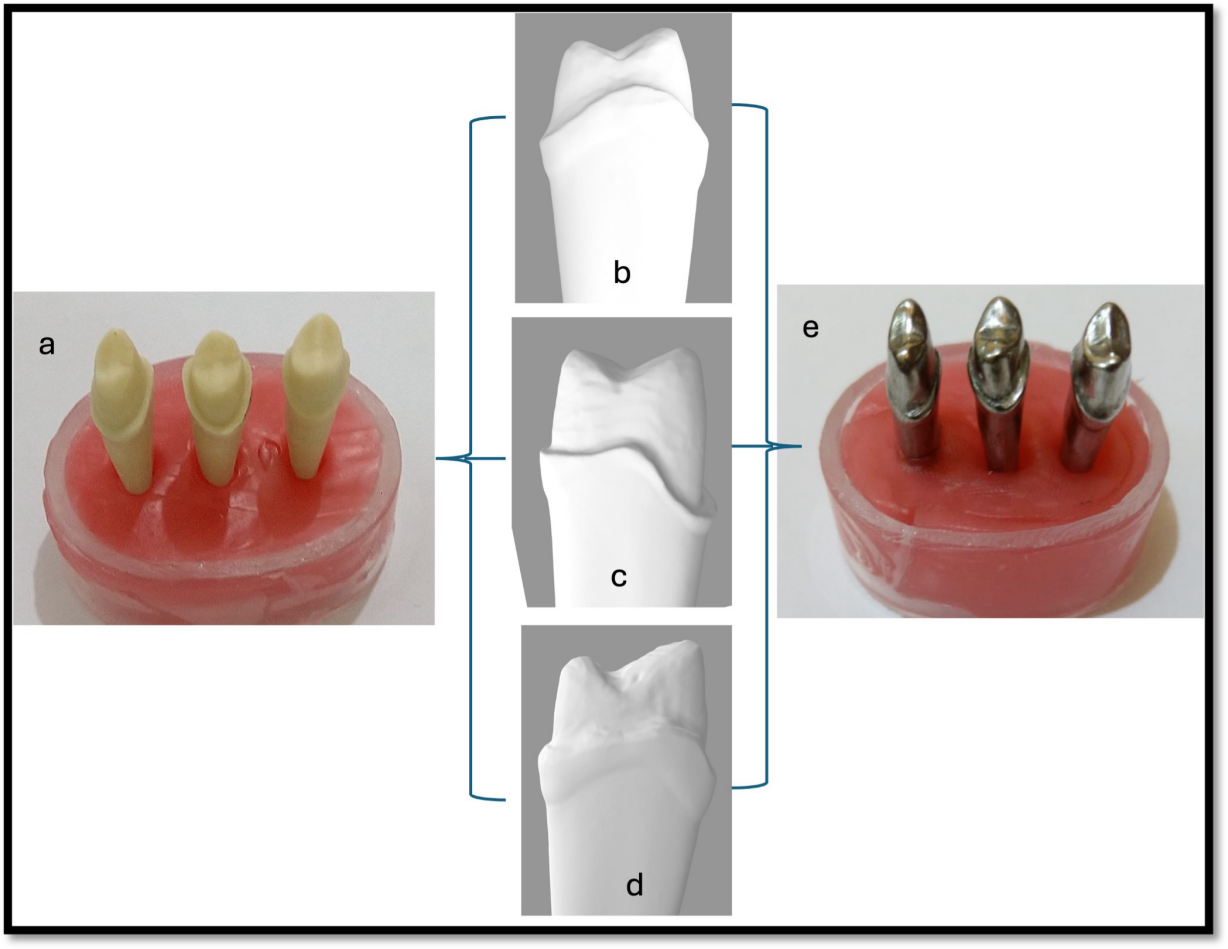
Steps for master metal die preparation. (A) Prepared typodont tooth #14 with finish line configuration (chamfer, rounded shoulder, rounded shoulder with bevel). (Center) Scanned virtual models of prepared typodont tooth #14 with finish line configuration (B) chamfer, (C) rounded shoulder, (D) rounded shoulder with bevel. (E) Master metal die of prepared typodont tooth #14.

A metal master model (cobalt-chromium; Wirobond C; BEGO GmbH; Bremen, Germany) was made from the tooth model after scanning it with a laboratory scanner and 3D printing it with castable resin (Fig. 2). The metal master dies prepared were duplicated, and study models were prepared using Type IV die stone (GC Fujirock EP; GC Europe) and coded as per their groups.

### Sample size and group division

The calculation was based on effect size determined by a previous study by Son, Lee & Lee (2021) in which they prepared 15 samples per technology and assessed the marginal and internal fit and intaglio surface trueness of temporary crowns fabricated with three techniques. To determine the sample size, an appropriate sample size was calculated as 30 per group, using power analysis (G*Power v3.1.9.4; Heinrich-Heine-Universität, Dusseldorf, Germany) based on the results of five pilot experiments with effect size [f] = 0.93; power = 0.95; actual power = 0.952; *α* = 0.05; numerator *df* = 5; denominator *df* = 21; groups = 6; number of covariates = 3. We acknowledge that, due to the three types of cervical preparation, the sample size per finish line sub-subgroup was calculated as 30.

Thus, 360 study models were created and categorised into two groups based on the type of intraoral scanner used for digital impressions. Group A, in which the intraoral scanner MEDIT i700 (Medit Songbuk, Kangwon-do, South Korea) was used and Group B, in which intraoral scanner TRIOS 3 (3Shape A/S, Niels Juels Gade 13,1059, Copenhagen, Denmark) was used (n = 360/group). The subgroups were made as Medit i700-SLA, Medit i700-DLP, TRIOS 3 -SLA and TRIOS 3-DLP (n = 180/subgroup). Further, these were divided into three sub-subgroups based on finish lines: CFL, RSFL, and RSBFL (n = 30 for each sub-subgroup) (Fig. 1).

### Fabrication of models and crown samples

Each study model of a particular group was initially scanned using the pre-decided intraoral scanner. The scanned image of the study model was stored in the DCM file format and later exported as a standard tessellation language (STL) file to the design software (CAD- 3shape design software) for the design and the fabrication of full coverage restorations. The virtual crowns without cementation space were designed and transferred to two 3D printers using two different 3D printing techniques. SLA 3D printer (Form 2 3D printer Formlabs Inc., Somerville, MA, USA) and DLP 3D printer (Cara Print 4.0 pro. Kulzer GmbH Hanau, Germany).

The castable resin (Formlabs Castable Wax Resin, Formlabs Inc., Somerville, MA, USA) was used to print the castable crowns using SLA and DLP-based printers (Dima Print Cast ruby, Kulzer GmbH Hanau, Germany). The castable crowns were printed under the condition of a 180° building angle with the occlusal surface facing the platform and a layer thickness of 25 µm in both printers.

**Figure 3:**
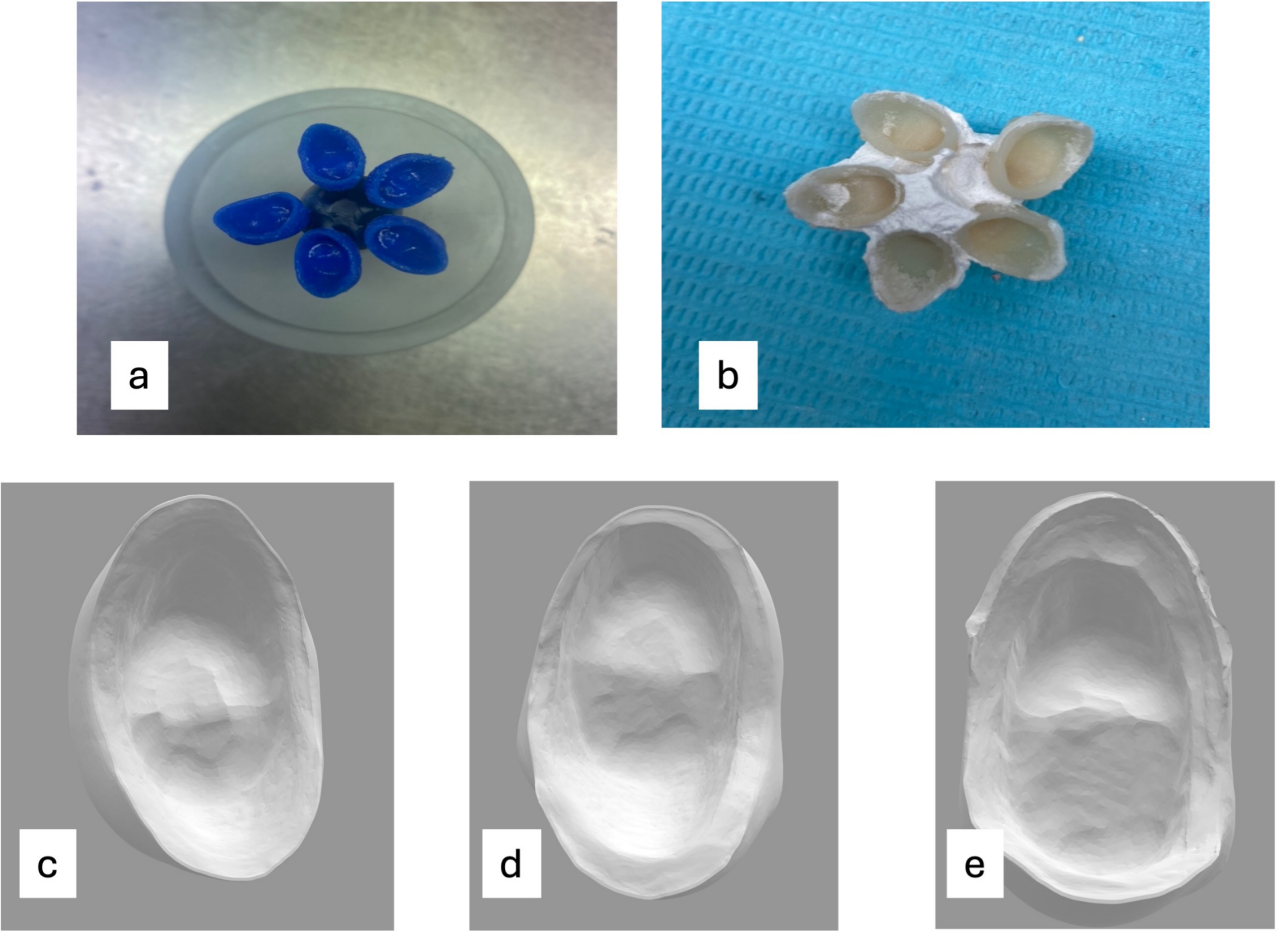
All ceramic crown fabrication. (A) 3D-printed castable resin patterns of designed crowns. (B) Pressed all ceramics crowns (IPS e.max Press lithium disilicate glass-ceramic (LS2) Ivoclar USA). (C) Representative image of the scanned intaglio surface of an all ceramic crown with a chamfer finish line. (D) Representative image of the scanned intaglio surface of an all ceramic crown with a rounded shoulder and finish line. (E) Representative image of the scanned intaglio surface of an all ceramic crown with a rounded shoulder with a bevel finish line.

These full veneer castable crowns were conventionally invested, and a refractory mould was made by using the lost wax technique (Elrashid et al., 2019; Alqahtani, 2017). Later, ceramics ingots (IPS e.max Press lithium disilicate glass-ceramic (LS2) Ivoclar USA) were pressed in the refractory mold to produce all ceramic crowns (Fig. 3). Each permanent crown so produced was correspondingly coded as per the study model. The minimal manipulation was done just to remove the investment material only, without any adjustments in the intaglio surface and to seat it over their respective study model.

### Measurement of marginal adaptation and internal fit

To determine the required measurements, the intaglio surface of each permanent crown was scanned using a laboratory scanner and aligned with the scan of prepared tooth surfaces using Medit design software. The superimposed images were assessed using software which provided the discrepancy between the superimposed images. The software used is Medit Design (Medit Corp., Seoul, Republic of Korea). Medit Design software is a complex tool that can analyse, align, measure (including distance, area, length, and angle), and compare 3D data. It offers a variety of tools to obtain the desired results, including “boolean”, “offset”, “smooth Surface”, “sculpting”, and many others.

**Figure 4:**
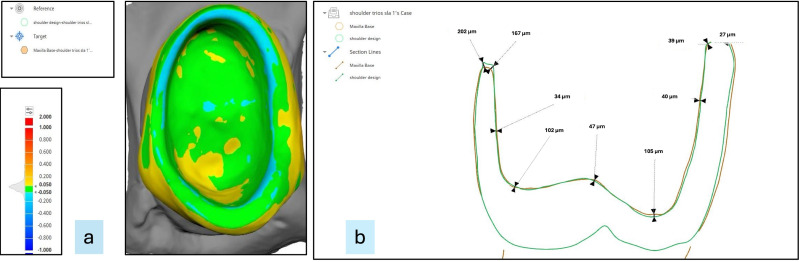
(A) Representative superimposed images of the intaglio surface of each permanent crown and prepared tooth surface. (B) Representative image showing the marginal adaptation and internal fit at various zones.

**Figure 5:**
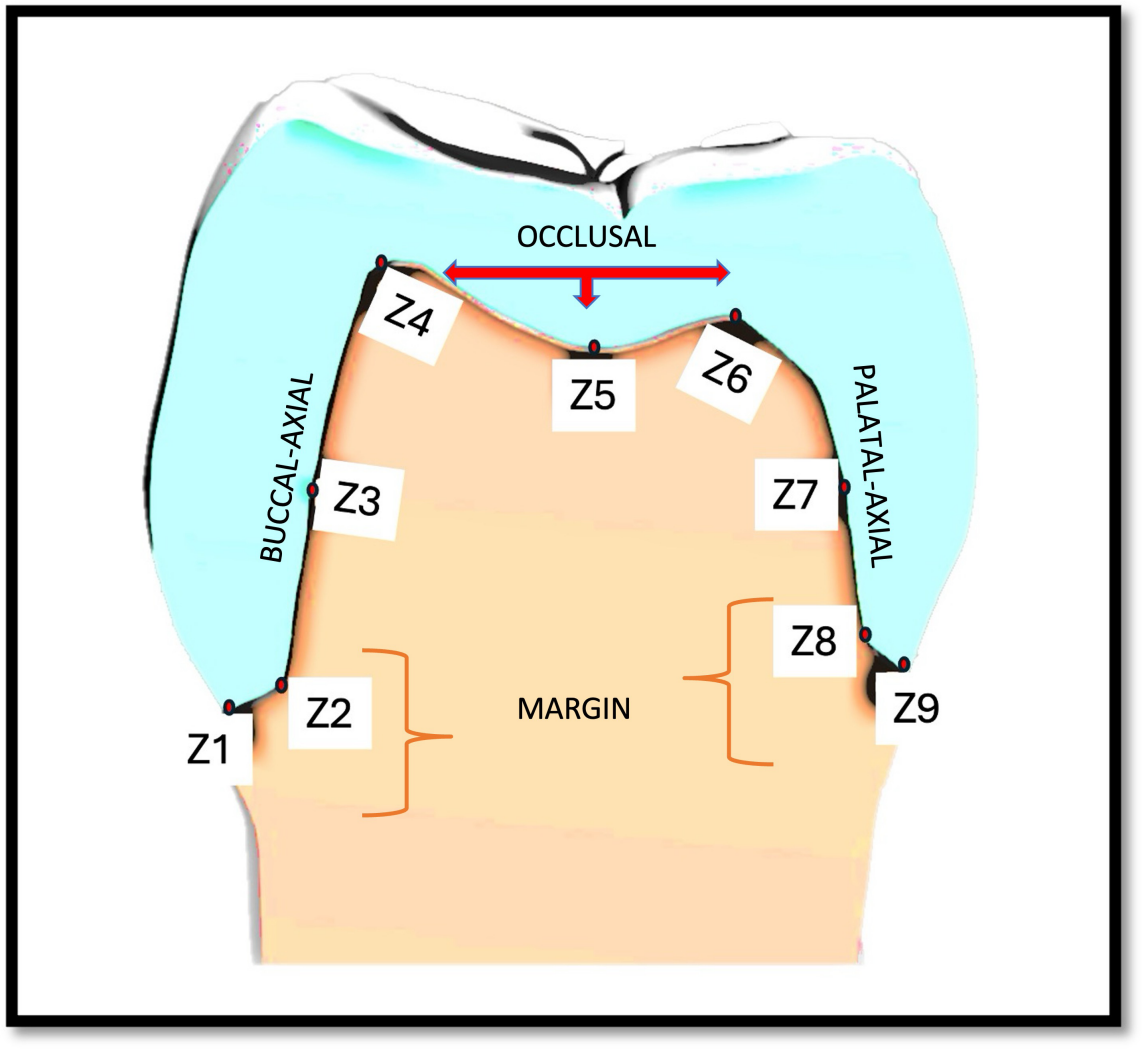
Representative image showing nine zones (Z1–Z9) on three selected surfaces, marginal, axial (buccal and palatal) and occlusal, for measurements.

To determine marginal adaptation and internal fit, measurements were recorded at nine zones (Z1–Z9) on the superimposed images (Fig. 4). These zones were compiled into—margin (Z1, Z2, Z8, Z9), axial {buccal axial (Z3), palatal axial (Z7)} and occlusal (Z4, Z5, Z6) (Fig. 5) of #14. Variations in fit across different areas (*e.g.*, margin, buccal, occlusal, palatal areas) of the crown can be attributed to the variations in the anatomy of the tooth, its preparation sensitivity, design of the crown, material, fabrication methods (precision of the printing or milling process) and scanning accuracy. By taking measurements at nine specific zones, all critical areas can be assessed. This method reduces the risk of missing localised discrepancies, resulting in a more dependable evaluation of the overall fit of the crown. This detailed analysis can help pinpoint specific areas where improvements in design or manufacturing could enhance fit quality. Utilising multi-zone measurements was suggested initially by Groten et al. (2000). It also allows for comparison with existing research, enhancing the robustness and relevance of the findings.

In the present study, the marginal discrepancy was measured at two points, one at a distance between the most extended point of the crown margin and the external marginal line of the prepared tooth and the second at the internal marginal line of the prepared tooth. For assessing internal discrepancy, the perpendicular distances from the external surface of the preparation to the internal surface of the crown were measured at the axial and occlusal surfaces (Al-Dwairi et al., 2019; Holmes et al., 1989; Guachetá et al., 2022). All measurements were recorded by the same operator trained and calibrated to use the software. To reduce the bias, each sample was measured twice, and the average of the two readings was calculated.

### Statistical analysis

The data was recorded, tabulated, and analysed using Jamovi (Version 2.3; https://www.jamovi.org). First, the normal distribution of the data was checked by the Shapiro–Wilk test. Later, the differences between groups were confirmed using a three-way analysis of variance (ANOVA), and pairwise comparison was done using the Tukey HSD test (*α* = 0.05).

## Results

The results of the study showed that the intraoral scanner, 3D printing techniques and finishing designs had affected marginal adaptation and internal fit of the permanent crown. The mean internal and marginal gaps were significantly influenced by the intraoral scanner (P = .000), fabrication method (P = .000), and finish line design (P = .000).

Group A-1 showed the lowest mean values for marginal gap and internal fit, particularly for RSBFL in each zone (Z1-Z9 49, 40, 42, 106, 48, 103, 35, 44, 47 respectively (in µm)); maximum for Group-B-2 for CFL (Z1-Z9 242, 233, 235, 299, 241, 296, 228, 237, 240 respectively (in µm)).

**Figure 6:**
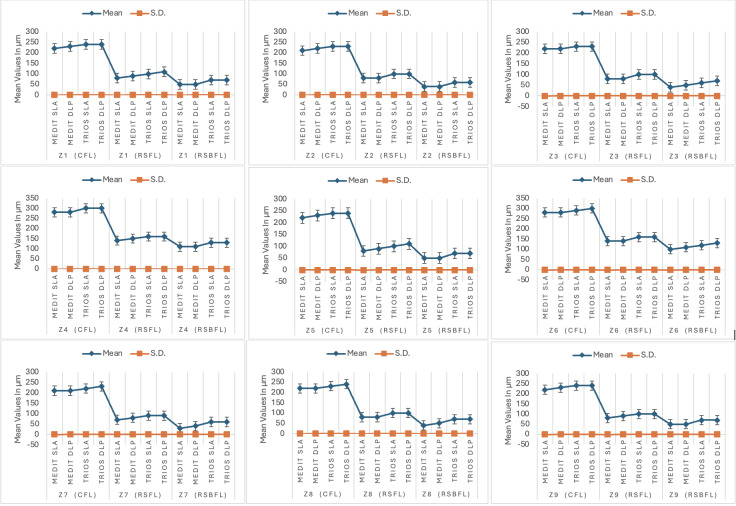
The mean and standard deviation of the measurements zonewise.

**Figure 7:**
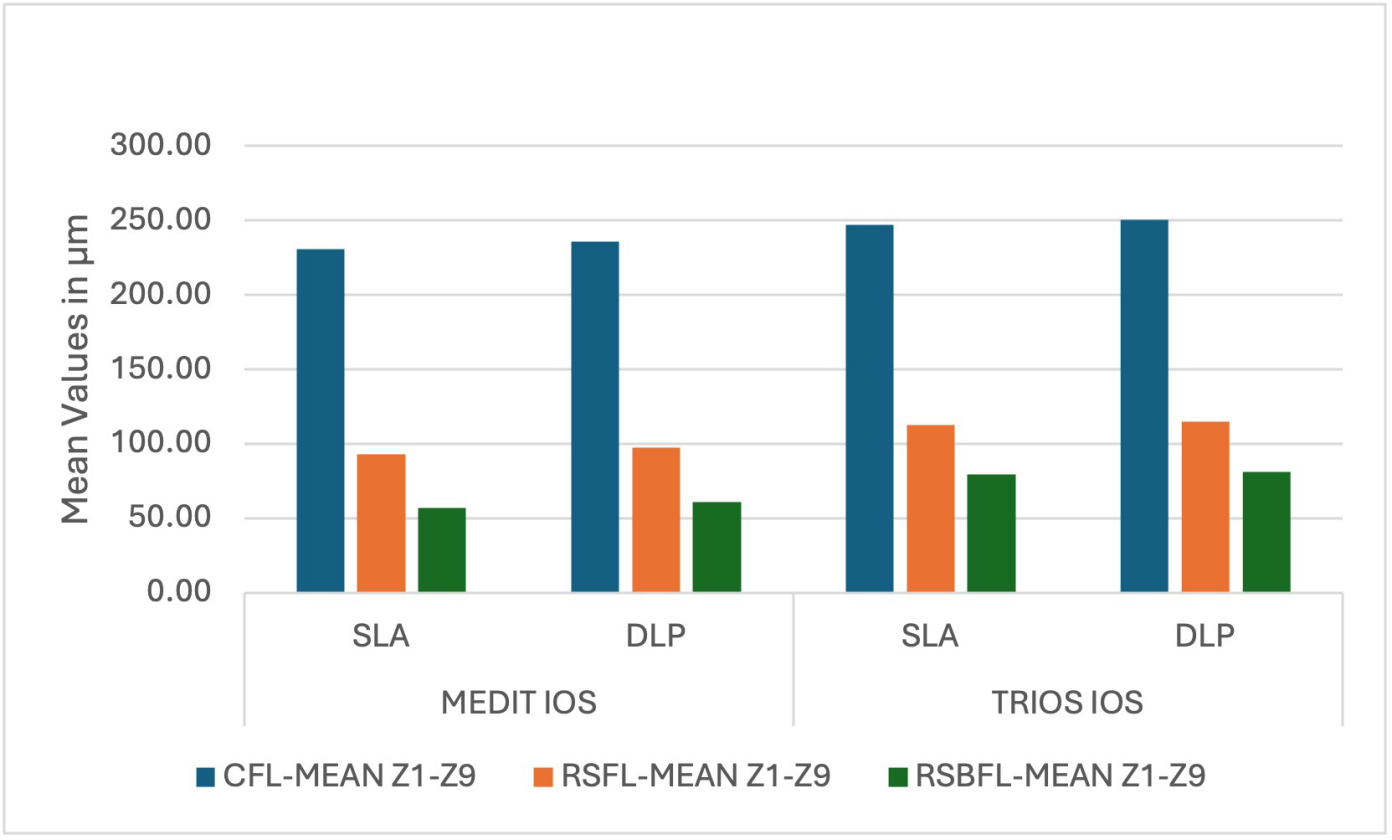
Intra-group and inter-group comparison of cumulative readings ((mean) for the marginal and internal fit of permanent crowns (all-ceramic)) with different finish lines manufactured using Medit and Trios IOS and SLA and DLP 3-D Pri.

**Figure 8:**
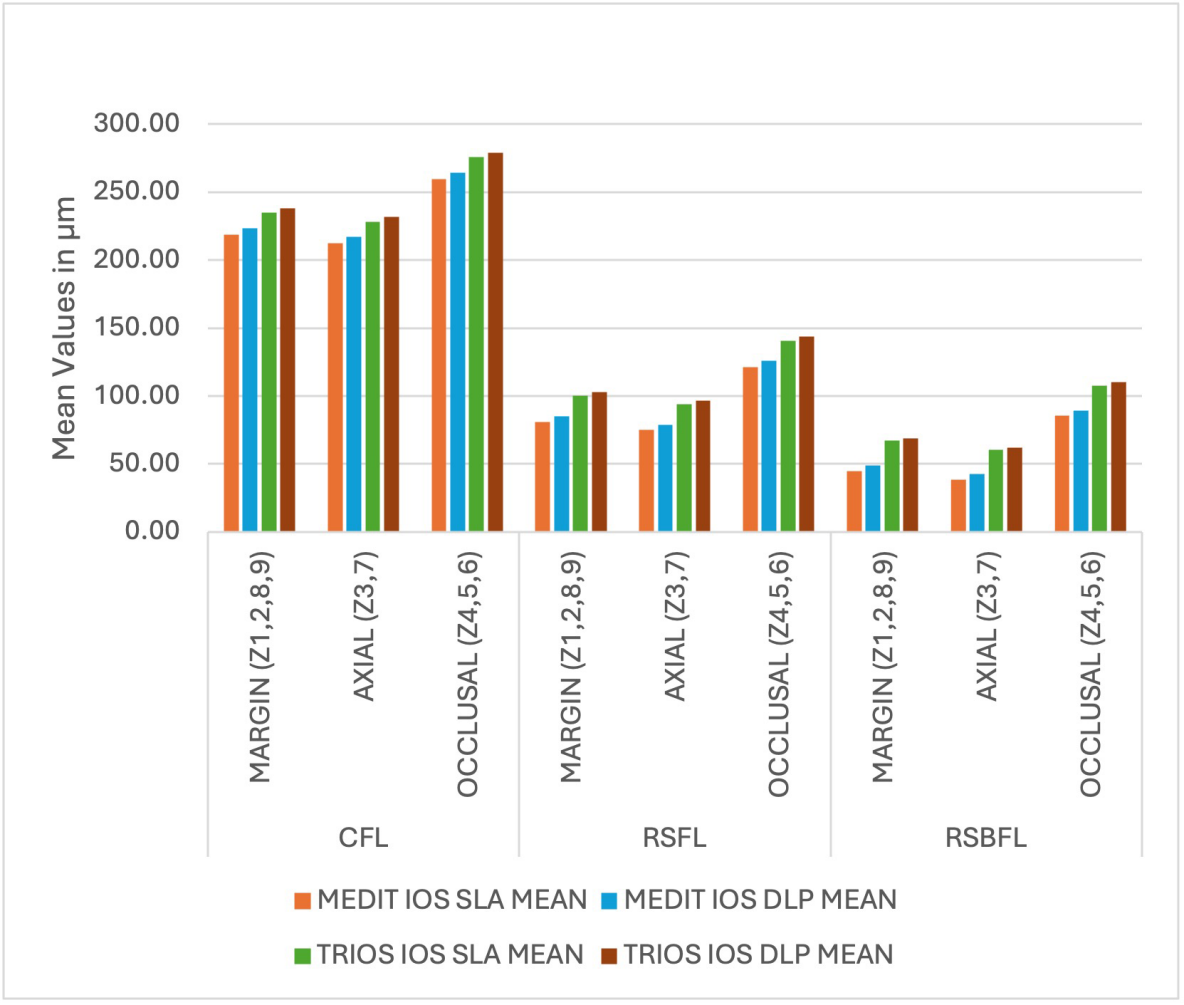
The comparison of cumulative readings ((mean) for the marginal and internal fit of permanent crowns (all-ceramic)) with the different finish lines at the margin, axial surface, and occlusal surface.

Group A-1 (Medit-SLA) showed minimum values for each finish line design with the lowest for RSBFL at zone Z2 40±0.001 (mean±SD) at the margin; Z7 30 ±0.001 (mean±SD) at axial surface, respectively and maximum for Group B-2 (TRIOS-DLP) for CFL at zone Z1 240±0.003 at the margin and Z4 300±0.002 (mean±SD) occlusal surface, respectively. Irrespective of the intraoral scanner and method of fabrication, the minimum gap was observed in RSBFL, followed by RSFL and CFL (Fig. 6). A similar result was recorded zone-wise, in each zone, the discrepancy between the crown and tooth was minimal in RSBFL, and a statistically significant difference was noted between the finish lines (Fig. 7). The maximum discrepancy was seen in the occlusal surface (Z4–Z6) followed by the axial surface (Z3, Z7) and minimum at margins (Z1, Z2, Z8, Z9) (Fig. 8).

The inter-group comparison and interaction for various independent variables (intraoral scanner (IOS), techniques and finishing designs) with the dependent variable (measurement values in µm) for marginal gap and the internal fit were assessed by three-way ANOVA. Even though the levene’s test for homogeneity of variances showed significant differences at the margin, occlusal and axial surfaces (*p* < 0.001) (Table 1), the robustness of ANOVA allow its use for statistical analysis, as previously mentioned by Tedesco et al. (2020) and Wang et al. (2017), specifically, when sample sizes are equal, as in the present study or nearly equal across groups. Three-way ANOVA can still yield valid results even in the presence of heterogeneity of variances. This robustness is particularly relevant in practical applications similar to present study where achieving perfect homogeneity is often challenging. The 3-way ANOVA showed that there was a significant difference between the various IOS (Medit and TRIOS), techniques (SLA and DLP) and finish lines (CFL, RSFL, RSBFL), but no significant difference was revealed when the interaction between the variables was assessed for each surface (Table 2) (Tables S1–S4).

**Table 1:** Levene’s test homogeneity of variances.

	Homogeneity of Variances Test (Levene’s)			
	**F**	**df1**	**df2**	***p***
MARGIN	11	11	348	<.001
B-AXIAL	348	11	348	<.001
OCCLUSAL	18.9	11	348	<.001
P-AXIAL	12	11	348	<.001

**Table 2:** Inter-group comparison and interaction effect of cumulative readings at margin, axial and occlusal surfaces of all ceramic crowns with different finish lines manufactured using Medit and Trios IOS and SLA and DLP 3-D Printing techniques–using 3.

ANOVA - MARGIN						ANOVA - B-AXIAL					ANOVA - OCCLUSAL					ANOVA - P-AXIAL				
** **	**Sum of squares**	**df**	**Mean square**	**F**	***p***	**Sum of squares**	**df**	**Mean square**	**F**	***p***	**Sum of squares**	**df**	**Mean square**	**F**	***p***	**Sum of squares**	**df**	**Mean square**	**F**	***p***
IOS	31,048.7	1	31,048.67	10,240.48	<.001	32,034.66	1	32,034.66	8,590.447	<.001	31,207.86	1	31,207.86	9,664.84	<.001	30,540.2	1	30,540.18	7,335.13	<.001
TECHNIQUE	1,770.7	1	1,770.67	584	<.001	1,399.92	1	1,399.92	375.405	<.001	2,542.96	1	2,542.96	787.54	<.001	1,166.1	1	1,166.08	280.07	<.001
FINISHLINE	1.97E+06	2	984,498.04	324,707.41	<.001	1.97E+06	2	984,171.47	263,916.444	<.001	1.96E+06	2	981,601.37	303,994.58	<.001	1.95E+06	2	975,757.52	234,357.09	<.001
IOS * TECHNIQUE * FINISHLINE	12.2	2	6.12	2.02	0.134	5.08	2	2.54	0.682	0.506	7.56	2	3.78	1.17	0.311	36.9	2	18.45	4.43	0.013

**Figure 9:**
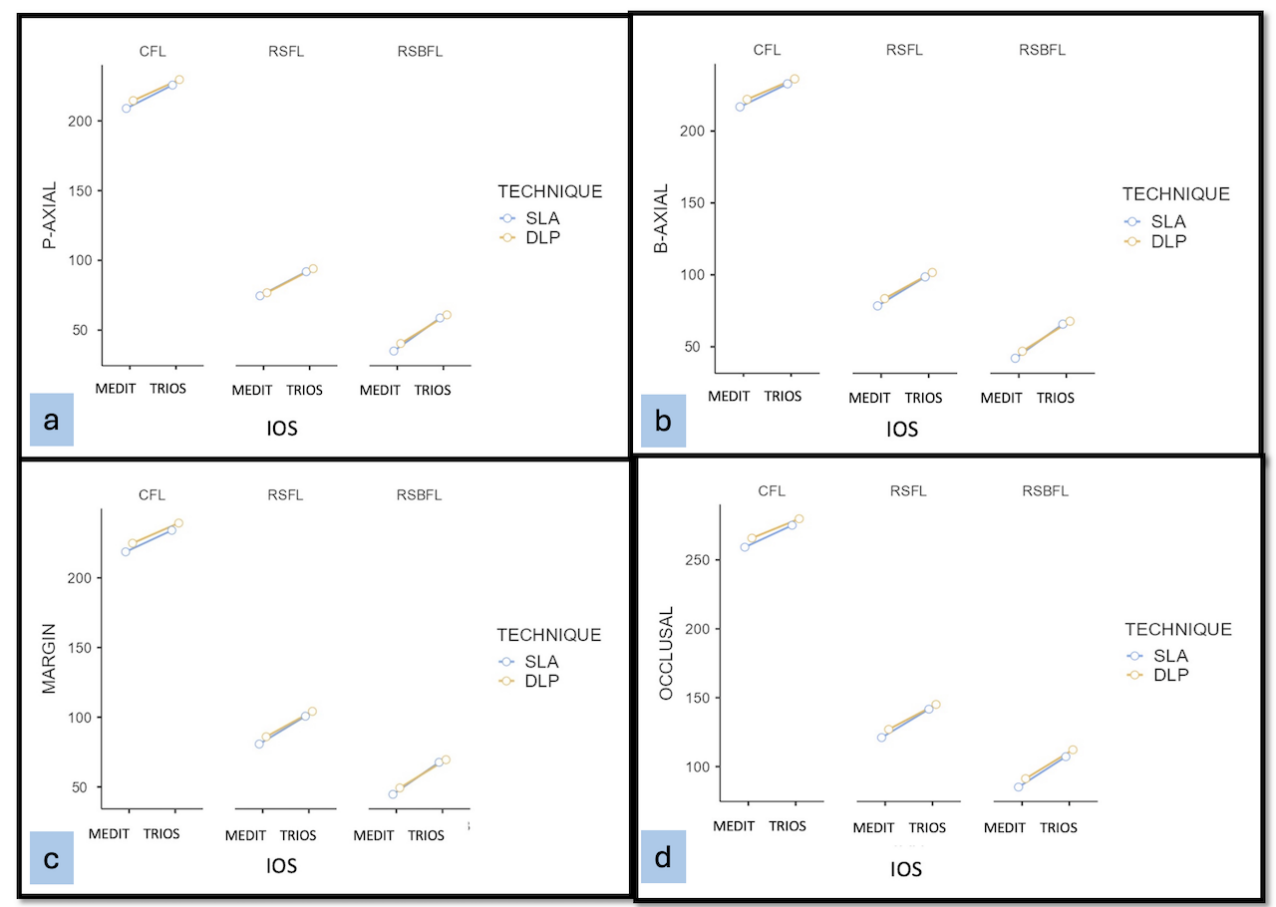
The estimated marginal means at P-axial surface. (A) P-axial surface; (B) B-axial; (C) margin; (D) occlusal.

On analysing the results, a statistically significant difference was found between the two groups (Medit and TRIOS), irrespective of the method of fabrication and finish lines studied. 3-way ANOVA followed by Tukey’s *post hoc* test in each surface between the groups’ a significant difference was noted. Also, there were significant differences between the SLA and DLP techniques in Medit-IOS (intra-group) Medit and TRIOS-IOS (inter-group). Overall, samples scanned with Medit and fabricated with SLA consistently showed minimal marginal gaps and improved internal fit, regardless of other factors, as shown in estimated marginal means plots for interaction effect (Fig. 9A–9D).

## Discussion

Constant developments in data gathering and production have augmented the need and popularity of digital work in dentistry (Alqahtani, 2017). It helps by making treatments easier and more comprehensible. Advanced digital dentistry techniques now allow for the fabrication of provisional and permanent crowns. Computer-aided design and computer-aided manufacturing (CAD/CAM) technology with intraoral scanning has evolved as a game changer in patient treatment modules. The subtractive technique (milling) has certain shortcomings, which include expensive devices (Park et al., 2016), higher making costs, and restrictions in complicated milling forms, particularly on the tissue surface (Peng et al., 2020), on the other hand, 3D printing is a recent technique and includes 3D printing of an entity by layering technique (Park et al., 2016; Joo et al., 2016; Kim et al., 2014; Munoz, Ramos & Dickinson, 2017). In the present study, the fabrication of all ceramic permanent crowns was done using two different intraoral scanners and SLA and DLP 3D printing technologies, and by using the superimposing technique, the marginal adaptation and internal fit were evaluated and compared. The null hypothesis was rejected, as there was a significant difference between the groups, with better fit and less discrepancy in Medit-IOS with the SLA 3D printing technique.

The present study is unique in its way as it compared the marginal adaptation and internal fit in permanent crowns made with different finish lines, scanned with different IOS and fabricated using two different 3D printing techniques.

The marginal gap is the measurement at the margin, and the internal gap is the perpendicular distance from the axial wall of the prepared tooth to the internal surface of the casting (Alharbi, Osman & Wismeijer, 2016). Though CAD/CAM has been a chosen technique in digital dentistry, there are major restrictions in processing a complex form, particularly the tissue surface of prostheses, due to the limited size and angles of the trimming tools (Alharbi, Osman & Wismeijer, 2016; Ishida & Miyasaka, 2016). Previous research mostly studied provisional crowns. A recent systematic review by Al Wadei et al. (2022) reported that the majority (*i.e.,* eight out of 13) of the studies documented that the least marginal inconsistencies were with the temporary crowns made by using 3D printing compared to CAD/CAM milling and conventional techniques (Al Wadei et al., 2022; Chaturvedi et al., 2020; Sidhom et al., 2022; Park et al., 2016; Aldahian et al., 2021).

Although there were variations in the marginal discrepancies in temporary restorations made using various materials and techniques, they were still less than the clinically acceptable limit of 120 µm (McLean & Von, 1971). In the current study, a similar outcome was observed with permanent crowns, where the discrepancy was less than 120 µm. Ostlund (1985) stipulate that the gap must be less than 50 µm. Although McLean & Von (1971) suggested that a gap of 120 µm should be the cut-off for clinical use, Sulaiman et al. (1997) claimed that 100 µm is adequate for clinical use. It may happen within the therapeutically appropriate range of up to 100–200 µm, according to Boening et al. (2000). From the figures, it can be concluded that the results of the current study’s measurements are satisfactory, achieving our study’s role in determining the dependability of CAD/CAM milling, SLA 3D printing, and DLP 3D printing in the production of temporary crowns.

In permanent crowns, the cement thickness is crucial. The suggested cement thickness ranges from no gap (Kim et al., 2017), 10 µm (Abdullah, Tsitrou & Pollington, 2016), and 15 µm with an extra 65 µm of vertical space and 50 µm (Anunmana, Charoenchitt & Asvanund, 2014), 30 µm (Wu & Wilson, 1994), 50 µm (Kokubo et al., 2005), 60 µm (Hoang et al., 2015), to 85 µm (Hoang et al., 2015) of horizontal spacing (Anunmana, Charoenchitt & Asvanund, 2014), respectively. To limit the likelihood of inadequate seating and pseudo gaps (Son et al., 2019; Al-Dwairi et al., 2019) and to prevent any errors in space estimation during the superimposition of scanned images, the samples in our investigation were designed without any gaps. Groten et al. (2000) advised using 20 to 25 measuring points per crown. In the current study, 30 samples—15 for each group, five for each type of finish line—were fabricated. To evaluate the fit, each sample was examined in nine zones with three randomly chosen points in each zone, resulting in 27 points in each sample and a total of 270 points for each type of finish line in each group (135 × 2 =270).

Various methods have been recommended for the determination of the fit of the restorations, including the scanning electron microscope (SEM) technique (difficulty in visualisation, technique-sensitive specimen preparation), replica method (problems in determining margins, tearing of layer of silicone, occurrence of defects in it and errors in the sectioning planes (Rahmé et al., 2008)), triple scan method (requires the agent which can create contrast (Holst et al., 2012)) and micro-CT technique (artefacts related to radiation, expensive (Sampaio et al., 2021)), in the present study the superimposition technique was implemented using auto-alignment software as it was simple, accurate, without any manual error, no loss of samples without any repletion of the procedures and it gives digitalised accurate measurement. The results of the present study showed that the discrepancy in the marginal adaptation and internal fit of the permanent crown made from the TRIOS-IOS with DLP 3D printing technique was maximum followed by TRIOS-IOS with SLA, Medit-DLP and the least misfit was noticed in Medit-SLA group. This was in association with Son, Lee & Lee (2021), in which the interim crowns made from the SLA technique had minimum discrepancy/ trueness compared to DLP and milled crowns (SLA (23.6 ±5.3 µm), DLP (29.0 ±3.6 µm), and milling groups (36.9 ±4.4 µm)). An earlier investigation into the potential use of 3D printing technology in a study on dental ceramic restorations examined the 3D trueness of zirconia crowns made utilising the technique (Methani, Revilla-León & Zandinejad, 2020). Another earlier study (Li et al., 2020) assessed the accuracy of zirconia crowns created using 3D gel deposition technology. According to the findings of this study, as well as those of earlier investigations, 3D printing technology is thought to have sufficient manufacturing accuracy for use in the medical field. The intaglio surface trueness/adaptation of interim crowns created using SLA technology (28.5 6.0 µm) (Yu, Da Son & Lee, 2021) and DLP technology (24.91 3.62 µm) (Lee et al., 2021) have been published in earlier studies. These findings demonstrated a comparable adaptation to the one found in the current investigation.

The present study compared the results of SLA and DLP of 3D printing technology, including the comparison of Medit and TRIOS-IOS technology. The results of this study showed that the permanent crown fabricated with SLA 3D printing technology revealed better results regardless of the evaluated region. The minimum misfit was in the margin area, followed by the axial and occlusal area. The justifiable reason would be the geometrical variations at various zones. Even though the technology for crown fabrication was the same, an automated relief function in dental CAD software for designing restorations uses a specialised algorithm to convert the image’s point clouds into a millable and printable one. The sections that are challenging to mill or print are combined into a simpler shape by this function. Even though this function is essential for practical usage, the approach may significantly deviate from the occlusal area and result in a mismatch (Mai, Lee & Lee, 2017). At the same time, in the relatively smooth axial surface and rounded margin area, the fitting was good (Lee, Lee & Lee, 2017).

The results of the present study contrast with the previous studies (Euán et al., 2014; Singh, Dhawan & Nautiyal, 2022; Grajower, Zuberi & Lewinstein, 1989) where DLP 3D printing produced better-fitting restorations. In the present study, the permanent crown produced by using the SLA technique showed better fit and adaptation.

Although SLA and DLP operate on the same theoretical foundation, the light source and method used to cure the layers of liquid resin vary, which significantly alters the final product. By scanning the laser beam inside the plane on the surface of a photosensitive material, SLA allows for sequential light exposure. Thus, the speed at which the laser beam is scanned, as well as the size of the illuminated area, dictate how long it takes to construct one slice of the structure. SLA and DLP both selectively cross-link layers of photo resin with the aid of light to produce free-standing objects. In contrast to SLA, each layer is simultaneously exposed, utilising a selectively veiled light source. The construction time is substantially shorter than that of SLA since the complete layer (slice) of the design is generated in a single exposure phase (Ligon et al., 2017). Such variations have a significant impact on the internal and marginal fit of manufactured dental crowns.

The three-dimensional CAD (3D-CAD) model is divided into several thin layers, and the manufacturing machinery uses this geometric data to generate each layer progressively until the object is completed (McLean & Von, 1971). This unique approach is based on this basic idea. And this is why there are fewer differences between 3D printing techniques. Although they are less frequent, differences can be caused by polymerization shrinkage that takes place when liquid photopolymer resin is cured with a light source (Park et al., 2019).

Regarding 3D printing systems, polymerization shrinkage is more pronounced where the extrusion sprues or supports—through which the resin is extruded in the desired manner are attached (Park et al., 2019). Because the sprues in the current investigation were linked to the occlusal surface, M4 would have had more polymerization shrinkage than other areas, which is why M4 recorded the highest values when the discrepancy was examined. CAD software is another factor that contributes to higher values at M4.

Irrespective of the construction method or material, the rounded shoulder with a bevel finish line had the lowest marginal discrepancy in our investigation, which was followed by the rounded shoulder and chamfer finish line. This may be because, as previously noted by Shillinburg, Jacobi & Brackett (1991) the closer the distance between the restoration margin and the tooth is, the more acutely the restoration margin ends. Additionally, results are consistent with research by Euán et al. (2014). Although this *in vitro* study showed that 3D printing enhances permanent crown fitting, clinical studies are still needed to confirm the current findings. Additionally, additional factors, including mechanical attributes, aesthetics, and cost-effectiveness, should be considered when using this technology in a therapeutic setting. Even though these computerised treatments have become more common in recent years, very few dentists still employ them. These methods can greatly simplify and expedite the work. Dental professionals can expedite the delivery of dental restorations by using a 3D printer or an in-house CAD/CAM milling machine. Dental laboratories should also be promoted for utilising these more recent technologies. The practitioners and technicians should communicate on one platform to do this.

### Limitations and future recommendations

Even though the study was performed with utmost accuracy, certain factors act as limitations to it. The *in-vitro* nature of the study, and the accuracy of 3D printed materials, would be influenced by the number of layers, layer intensity, total thickness, UV intensity, post-processing method, number, and placement of support structures (Kim et al., 2017; Hoang et al., 2015; Al Deeb et al., 2020; Savencu, Şerban & Porojan, 2020) were not taken into consideration. Also, the lack of a control group using a conventional technique, such as milling or wax pressing, would have allowed for a more robust comparison of the new 3D printing techniques with established methods. It is recommended that future studies take these points into consideration or further exploration to assess comparative efficacy.

## Conclusions

Based on the findings of this *in vitro* study, the following conclusions were drawn:

1. All-ceramic crowns made using the Medit intraoral scanner and SLA 3D printer with a rounded shoulder finish line exhibited the best marginal and internal fit.
2. The crowns produced with the SLA 3D printer had better marginal and internal fit compared to the crowns produced with the DLP 3D printer.
3. The crowns with rounded shoulder finish lines with bevels had better marginal and internal fit, irrespective of the technique of fabrication used.

##  Supplemental Information

10.7717/peerj.19117/supp-1 Supplemental Information 1 Raw data

10.7717/peerj.19117/supp-2 Supplemental Information 2 Supplementary Tables

## Abbreviations

3D: 3 Dimensional
CAD-CAM: Computer-Aided Design and Computer-Aided Manufacturing
STL: Standard tessellation language
DICOM: Digital Imaging and Communications in Medicine
FDM: Fused deposition modelling
SLA: Stereolithography
SLS: Selective laser sintering
SLM: Selective laser melting
PBP: Powder binder printers
DLP: Digital light processing
CFL: Chamfer Finish Line
RSFL: Rounded shoulder Finish line
RSBFL: Rounded shoulder with a bevel Finish line
Z: ZONE

## Additional Information and Declarations

### Data Availability

The following information was supplied regarding data availability: The raw data is available in the Supplemental File.
