# Supplementary material for: Advanced superimposition method to evaluate the marginal and internal fit of ceramic crowns fabricated using heat pressing techniques
Source: PeerJ. 2025 Apr 3;13:e19117. doi: 10.7717/peerj.19117 (PMC11972566; doi:10.7717/peerj.19117)
Supplement: Supplemental Information 1 [file peerj-13-19117-s001.pdf]

S.NO.	IOS	TECH	FL	MARGIN	BA	O	PA
1	MEDIT -i700	SLA	CFL	217.80	215.00	258.67	208.00
2	MEDIT -i700	SLA	CFL	219.18	215.90	259.97	208.80
3	MEDIT -i700	SLA	CFL	218.95	216.20	259.97	209.30
4	MEDIT -i700	SLA	CFL	218.78	216.10	259.77	209.10
5	MEDIT -i700	SLA	CFL	218.55	215.90	259.57	208.90
6	MEDIT -i700	SLA	CFL	218.86	215.53	258.92	208.15
7	MEDIT -i700	SLA	CFL	218.48	216.21	258.75	208.67
8	MEDIT -i700	SLA	CFL	218.40	217.99	258.18	208.92
9	MEDIT -i700	SLA	CFL	219.22	217.59	259.26	208.33
10	MEDIT -i700	SLA	CFL	218.84	215.45	259.58	209.45
11	MEDIT -i700	SLA	CFL	217.87	215.32	259.52	208.76
12	MEDIT -i700	SLA	CFL	219.07	216.12	259.82	209.58
13	MEDIT -i700	SLA	CFL	218.46	218.18	259.85	209.12
14	MEDIT -i700	SLA	CFL	219.60	215.28	259.51	208.59
15	MEDIT -i700	SLA	CFL	217.28	216.64	259.66	209.77
16	MEDIT -i700	SLA	CFL	218.05	217.50	258.25	208.53
17	MEDIT -i700	SLA	CFL	218.57	215.18	259.16	209.03
18	MEDIT -i700	SLA	CFL	219.19	215.84	259.31	208.47
19	MEDIT -i700	SLA	CFL	219.30	218.04	259.68	209.91
20	MEDIT -i700	SLA	CFL	217.43	218.56	259.12	208.64
21	MEDIT -i700	SLA	CFL	217.78	218.29	259.47	208.89
22	MEDIT -i700	SLA	CFL	219.69	216.10	259.90	208.29
23	MEDIT -i700	SLA	CFL	217.36	218.70	258.43	209.31
24	MEDIT -i700	SLA	CFL	219.68	217.99	259.03	209.18
25	MEDIT -i700	SLA	CFL	217.39	217.90	259.66	208.25
26	MEDIT -i700	SLA	CFL	219.77	218.06	259.07	209.54
27	MEDIT -i700	SLA	CFL	219.21	215.26	259.73	208.72
28	MEDIT -i700	SLA	CFL	218.92	217.49	258.24	208.44
29	MEDIT -i700	SLA	CFL	219.30	218.71	258.56	209.67
30	MEDIT -i700	SLA	CFL	219.53	217.84	259.40	208.21
31	MEDIT -i700	SLA	RSFL	79.80	77.00	120.67	70.00
32	MEDIT -i700	SLA	RSFL	81.18	77.90	121.60	71.20
33	MEDIT -i700	SLA	RSFL	81.03	79.80	121.97	78.90
34	MEDIT -i700	SLA	RSFL	80.90	78.10	121.77	71.10
35	MEDIT -i700	SLA	RSFL	80.70	77.90	121.57	70.90
36	MEDIT -i700	SLA	RSFL	80.22	78.42	120.45	71.52
37	MEDIT -i700	SLA	RSFL	80.30	77.88	121.12	77.34
38	MEDIT -i700	SLA	RSFL	80.94	79.14	120.83	75.91
39	MEDIT -i700	SLA	RSFL	81.68	78.67	121.67	72.18
40	MEDIT -i700	SLA	RSFL	81.99	77.55	120.91	76.63
41	MEDIT -i700	SLA	RSFL	80.12	79.31	121.56	73.47
42	MEDIT -i700	SLA	RSFL	79.29	78.25	120.74	74.29
43	MEDIT -i700	SLA	RSFL	80.34	77.79	121.03	78.05
44	MEDIT -i700	SLA	RSFL	80.59	79.23	120.26	75.62
45	MEDIT -i700	SLA	RSFL	80.91	78.81	121.21	71.79
46	MEDIT -i700	SLA	RSFL	79.93	78.53	120.39	77.11
47	MEDIT -i700	SLA	RSFL	80.23	78.16	121.87	72.56
48	MEDIT -i700	SLA	RSFL	81.93	77.68	120.62	76.41
49	MEDIT -i700	SLA	RSFL	79.42	79.56	121.42	73.95
50	MEDIT -i700	SLA	RSFL	80.83	77.45	120.55	75.28
51	MEDIT -i700	SLA	RSFL	81.10	79.49	121.36	77.73
52	MEDIT -i700	SLA	RSFL	81.78	78.98	120.11	74.83
53	MEDIT -i700	SLA	RSFL	80.70	78.37	121.29	71.92
54	MEDIT -i700	SLA	RSFL	81.89	77.90	120.51	78.43
55	MEDIT -i700	SLA	RSFL	79.17	79.05	121.09	73.29
56	MEDIT -i700	SLA	RSFL	81.56	78.62	120.64	74.06
57	MEDIT -i700	SLA	RSFL	79.91	77.33	121.95	76.22
58	MEDIT -i700	SLA	RSFL	81.98	78.09	120.77	72.64
59	MEDIT -i700	SLA	RSFL	80.53	79.68	121.15	75.48
60	MEDIT -i700	SLA	RSFL	81.58	78.71	120.28	78.17
61	MEDIT -i700	SLA	RSBFL	43.80	41.00	84.67	34.00
62	MEDIT -i700	SLA	RSBFL	44.98	42.20	85.87	35.20
63	MEDIT -i700	SLA	RSBFL	45.10	42.30	85.90	35.30
64	MEDIT -i700	SLA	RSBFL	44.90	42.40	85.77	35.10
65	MEDIT -i700	SLA	RSBFL	44.70	41.90	85.50	34.90
66	MEDIT -i700	SLA	RSBFL	44.31	41.86	84.75	34.56
67	MEDIT -i700	SLA	RSBFL	45.45	42.12	85.12	35.12
68	MEDIT -i700	SLA	RSBFL	44.62	41.77	84.56	34.87
69	MEDIT -i700	SLA	RSBFL	45.55	42.56	85.28	35.41
70	MEDIT -i700	SLA	RSBFL	43.29	41.34	84.92	34.75
71	MEDIT -i700	SLA	RSBFL	44.70	42.84	85.64	35.29
72	MEDIT -i700	SLA	RSBFL	44.70	41.61	84.41	34.68
73	MEDIT -i700	SLA	RSBFL	45.19	42.02	85.53	35.84
74	MEDIT -i700	SLA	RSBFL	43.99	41.93	84.23	34.92
75	MEDIT -i700	SLA	RSBFL	43.30	42.23	85.76	35.63
76	MEDIT -i700	SLA	RSBFL	45.33	41.79	84.60	34.53
77	MEDIT -i700	SLA	RSBFL	44.51	42.47	85.48	35.22
78	MEDIT -i700	SLA	RSBFL	45.13	42.05	84.87	34.29
79	MEDIT -i700	SLA	RSBFL	45.97	41.51	85.35	35.77
80	MEDIT -i700	SLA	RSBFL	45.73	42.11	84.16	34.11
81	MEDIT -i700	SLA	RSBFL	44.32	42.71	85.81	35.56
82	MEDIT -i700	SLA	RSBFL	43.27	41.28	84.68	34.63
83	MEDIT -i700	SLA	RSBFL	43.71	42.38	85.24	35.09
84	MEDIT -i700	SLA	RSBFL	45.08	41.42	84.31	34.82
85	MEDIT -i700	SLA	RSBFL	43.20	42.18	85.09	35.38
86	MEDIT -i700	SLA	RSBFL	43.74	41.66	84.82	34.94
87	MEDIT -i700	SLA	RSBFL	45.97	42.85	85.43	35.18
88	MEDIT -i700	SLA	RSBFL	45.26	41.93	84.97	34.41
89	MEDIT -i700	SLA	RSBFL	45.28	42.30	85.61	35.67
90	MEDIT -i700	SLA	RSBFL	44.14	41.56	84.50	34.37
91	MEDIT -i700	DLP	CFL	221.40	218.60	262.27	211.60
92	MEDIT -i700	DLP	CFL	222.80	220.00	263.33	213.00
93	MEDIT -i700	DLP	CFL	223.10	220.10	263.97	213.30
94	MEDIT -i700	DLP	CFL	222.70	219.90	263.57	212.50
95	MEDIT -i700	DLP	CFL	228.40	225.60	269.27	218.60
96	MEDIT -i700	DLP	CFL	227.03	220.34	264.73	213.47
97	MEDIT -i700	DLP	CFL	227.27	223.12	267.58	217.02
98	MEDIT -i700	DLP	CFL	221.59	218.79	262.47	212.89
99	MEDIT -i700	DLP	CFL	225.88	224.56	268.32	215.61
100	MEDIT -i700	DLP	CFL	221.25	221.45	265.19	214.34
101	MEDIT -i700	DLP	CFL	225.05	223.88	267.81	217.76
102	MEDIT -i700	DLP	CFL	221.25	221.92	263.64	213.22
103	MEDIT -i700	DLP	CFL	226.97	225.14	268.01	216.11
104	MEDIT -i700	DLP	CFL	224.93	219.63	264.12	211.94
105	MEDIT -i700	DLP	CFL	226.12	224.73	269.34	217.44
106	MEDIT -i700	DLP	CFL	225.41	220.58	262.93	213.68
107	MEDIT -i700	DLP	CFL	227.85	223.41	266.87	214.90
108	MEDIT -i700	DLP	CFL	221.47	221.09	267.11	212.57
109	MEDIT -i700	DLP	CFL	225.14	225.27	265.58	216.74
110	MEDIT -i700	DLP	CFL	226.79	219.47	268.57	211.73
111	MEDIT -i700	DLP	CFL	223.25	222.36	263.29	217.38
112	MEDIT -i700	DLP	CFL	224.89	218.62	269.43	213.59
113	MEDIT -i700	DLP	CFL	226.83	224.09	264.95	215.82
114	MEDIT -i700	DLP	CFL	224.78	222.17	267.44	212.05
115	MEDIT -i700	DLP	CFL	227.07	225.03	262.25	217.21
116	MEDIT -i700	DLP	CFL	225.07	220.97	266.53	214.27
117	MEDIT -i700	DLP	CFL	228.57	224.25	269.18	216.52
118	MEDIT -i700	DLP	CFL	224.41	219.84	263.78	213.16
119	MEDIT -i700	DLP	CFL	223.76	223.53	265.36	214.81
120	MEDIT -i700	DLP	CFL	225.03	221.61	268.88	212.43
121	MEDIT -i700	DLP	RSFL	82.90	80.10	123.77	73.10
122	MEDIT -i700	DLP	RSFL	84.13	81.50	125.17	74.50
123	MEDIT -i700	DLP	RSFL	84.53	81.80	125.47	74.80
124	MEDIT -i700	DLP	RSFL	84.20	81.40	125.07	74.40
125	MEDIT -i700	DLP	RSFL	89.90	87.10	130.77	80.10
126	MEDIT -i700	DLP	RSFL	84.15	85.24	127.54	77.63
127	MEDIT -i700	DLP	RSFL	84.71	82.56	124.62	73.92
128	MEDIT -i700	DLP	RSFL	88.95	86.72	129.87	79.48
129	MEDIT -i700	DLP	RSFL	84.91	80.93	126.43	74.56
130	MEDIT -i700	DLP	RSFL	88.32	84.18	128.15	78.24
131	MEDIT -i700	DLP	RSFL	83.83	81.47	123.77	75.67
132	MEDIT -i700	DLP	RSFL	87.31	86.35	125.91	79.13
133	MEDIT -i700	DLP	RSFL	87.96	81.92	130.32	75.31
134	MEDIT -i700	DLP	RSFL	83.53	85.68	127.89	80.01
135	MEDIT -i700	DLP	RSFL	85.24	84.75	129.03	74.92
136	MEDIT -i700	DLP	RSFL	87.84	81.15	124.79	76.58
137	MEDIT -i700	DLP	RSFL	85.21	86.02	128.49	79.29
138	MEDIT -i700	DLP	RSFL	89.12	80.57	125.36	77.21
139	MEDIT -i700	DLP	RSFL	88.33	82.93	130.14	73.78
140	MEDIT -i700	DLP	RSFL	87.03	85.40	124.21	78.92
141	MEDIT -i700	DLP	RSFL	86.30	81.63	126.88	76.16
142	MEDIT -i700	DLP	RSFL	89.93	87.25	127.12	74.47
143	MEDIT -i700	DLP	RSFL	83.66	80.76	130.68	80.56
144	MEDIT -i700	DLP	RSFL	84.53	84.47	125.58	77.89
145	MEDIT -i700	DLP	RSFL	83.32	86.11	129.47	75.83
146	MEDIT -i700	DLP	RSFL	85.77	82.48	123.91	78.14
147	MEDIT -i700	DLP	RSFL	87.52	85.91	128.91	77.39
148	MEDIT -i700	DLP	RSFL	82.32	81.29	124.58	74.68
149	MEDIT -i700	DLP	RSFL	89.01	80.64	130.21	79.77
150	MEDIT -i700	DLP	RSFL	85.17	87.14	126.15	76.73
151	MEDIT -i700	DLP	RSBFL	46.40	43.60	87.27	36.60
152	MEDIT -i700	DLP	RSBFL	47.80	45.00	88.67	38.00
153	MEDIT -i700	DLP	RSBFL	48.10	45.20	88.90	38.30
154	MEDIT -i700	DLP	RSBFL	47.70	44.90	88.40	37.90
155	MEDIT -i700	DLP	RSBFL	53.40	50.60	94.27	43.60
156	MEDIT -i700	DLP	RSBFL	48.28	47.12	92.38	41.72
157	MEDIT -i700	DLP	RSBFL	48.21	44.83	88.72	37.56
158	MEDIT -i700	DLP	RSBFL	48.74	49.67	91.56	43.14
159	MEDIT -i700	DLP	RSBFL	47.93	45.99	89.91	38.29
160	MEDIT -i700	DLP	RSBFL	53.66	48.52	92.47	42.91
161	MEDIT -i700	DLP	RSBFL	52.85	46.34	90.11	41.08
162	MEDIT -i700	DLP	RSBFL	53.01	45.71	94.03	39.63
163	MEDIT -i700	DLP	RSBFL	48.33	49.08	89.33	43.67
164	MEDIT -i700	DLP	RSBFL	47.85	44.27	93.81	37.92
165	MEDIT -i700	DLP	RSBFL	49.74	49.41	90.77	42.49
166	MEDIT -i700	DLP	RSBFL	48.51	46.95	94.55	41.53
167	MEDIT -i700	DLP	RSBFL	51.00	48.24	91.64	38.71
168	MEDIT -i700	DLP	RSBFL	48.15	45.18	88.53	43.25
169	MEDIT -i700	DLP	RSBFL	52.57	47.67	92.19	40.83
170	MEDIT -i700	DLP	RSBFL	46.42	44.91	93.24	37.47
171	MEDIT -i700	DLP	RSBFL	50.13	50.22	90.93	42.15
172	MEDIT -i700	DLP	RSBFL	48.35	46.76	91.04	41.39
173	MEDIT -i700	DLP	RSBFL	46.62	49.87	93.12	38.18
174	MEDIT -i700	DLP	RSBFL	47.00	44.52	89.67	43.76
175	MEDIT -i700	DLP	RSBFL	47.48	47.81	94.29	40.24
176	MEDIT -i700	DLP	RSBFL	51.32	45.42	92.62	37.88
177	MEDIT -i700	DLP	RSBFL	52.17	49.14	88.86	42.62
178	MEDIT -i700	DLP	RSBFL	48.87	46.57	93.50	40.98
179	MEDIT -i700	DLP	RSBFL	46.46	48.83	90.36	39.12
180	MEDIT -i700	DLP	RSBFL	51.10	44.65	91.82	41.01

S.NO.	IOS	TECH	FL	MARGIN	BA	O	PA
1	TRIOS 3	SLA	CFL	232.80	230.00	273.67	223.00
2	TRIOS 3	SLA	CFL	233.75	231.10	275.00	224.10
3	TRIOS 3	SLA	CFL	234.05	231.50	274.97	224.50
4	TRIOS 3	SLA	CFL	238.80	233.00	279.67	229.00
5	TRIOS 3	SLA	CFL	235.30	233.00	276.67	221.00
6	TRIOS 3	SLA	CFL	233.47	232.47	275.34	224.15
7	TRIOS 3	SLA	CFL	234.12	234.12	274.67	227.34
8	TRIOS 3	SLA	CFL	232.68	231.68	273.89	221.87
9	TRIOS 3	SLA	CFL	234.85	235.31	276.22	229.52
10	TRIOS 3	SLA	CFL	233.21	232.92	275.01	223.67
11	TRIOS 3	SLA	CFL	235.13	233.53	274.56	228.41
12	TRIOS 3	SLA	CFL	234.33	230.89	275.77	224.92
13	TRIOS 3	SLA	CFL	232.92	234.85	273.45	229.11
14	TRIOS 3	SLA	CFL	235.45	231.21	276.12	222.58
15	TRIOS 3	SLA	CFL	233.87	234.68	275.56	227.63
16	TRIOS 3	SLA	CFL	234.57	232.56	273.93	224.81
17	TRIOS 3	SLA	CFL	232.11	235.09	276.68	225.74
18	TRIOS 3	SLA	CFL	233.53	230.43	274.32	228.26
19	TRIOS 3	SLA	CFL	235.31	233.19	275.44	222.99
20	TRIOS 3	SLA	CFL	234.08	231.78	274.91	228.73
21	TRIOS 3	SLA	CFL	232.45	234.23	273.61	224.39
22	TRIOS 3	SLA	CFL	233.14	233.61	275.23	227.18
23	TRIOS 3	SLA	CFL	234.74	232.35	276.47	226.44
24	TRIOS 3	SLA	CFL	233.61	234.77	274.23	221.31
25	TRIOS 3	SLA	CFL	235.02	230.72	276.09	229.25
26	TRIOS 3	SLA	CFL	234.62	235.04	275.12	223.42
27	TRIOS 3	SLA	CFL	232.56	233.14	274.78	228.05
28	TRIOS 3	SLA	CFL	233.72	231.59	273.82	225.88
29	TRIOS 3	SLA	CFL	234.27	234.41	275.68	226.79
30	TRIOS 3	SLA	CFL	235.19	232.18	274.15	224.53
31	TRIOS 3	SLA	RSFL	97.80	95.00	138.67	88.00
32	TRIOS 3	SLA	RSFL	98.90	96.10	139.77	89.10
33	TRIOS 3	SLA	RSFL	99.55	96.50	140.07	89.50
34	TRIOS 3	SLA	RSFL	103.80	101.00	144.67	94.00
35	TRIOS 3	SLA	RSFL	100.80	98.00	141.33	91.00
36	TRIOS 3	SLA	RSFL	101.45	97.52	142.63	92.34
37	TRIOS 3	SLA	RSFL	98.72	100.34	139.84	90.68
38	TRIOS 3	SLA	RSFL	102.18	96.71	141.27	94.12
39	TRIOS 3	SLA	RSFL	100.36	101.12	143.55	91.45
40	TRIOS 3	SLA	RSFL	98.91	97.89	140.92	93.21
41	TRIOS 3	SLA	RSFL	102.65	99.45	141.79	89.76
42	TRIOS 3	SLA	RSFL	101.13	95.84	142.41	94.58
43	TRIOS 3	SLA	RSFL	99.84	100.76	139.37	90.92
44	TRIOS 3	SLA	RSFL	100.59	96.38	144.15	92.56
45	TRIOS 3	SLA	RSFL	103.27	101.43	140.61	91.88
46	TRIOS 3	SLA	RSFL	97.55	97.65	143.12	94.35
47	TRIOS 3	SLA	RSFL	101.78	100.03	141.04	89.03
48	TRIOS 3	SLA	RSFL	100.04	98.52	139.92	93.76
49	TRIOS 3	SLA	RSFL	102.21	101.21	144.28	92.09
50	TRIOS 3	SLA	RSFL	98.54	96.90	141.16	90.24
51	TRIOS 3	SLA	RSFL	103.14	100.89	142.87	94.67
52	TRIOS 3	SLA	RSFL	101.62	98.14	140.45	91.31
53	TRIOS 3	SLA	RSFL	99.76	99.78	143.72	92.81
54	TRIOS 3	SLA	RSFL	102.48	95.55	139.23	89.57
55	TRIOS 3	SLA	RSFL	100.23	101.67	142.55	93.49
56	TRIOS 3	SLA	RSFL	103.06	97.01	141.34	90.51
57	TRIOS 3	SLA	RSFL	97.88	100.22	144.02	91.14
58	TRIOS 3	SLA	RSFL	101.97	99.13	140.10	94.21
59	TRIOS 3	SLA	RSFL	99.12	96.53	143.93	92.15
60	TRIOS 3	SLA	RSFL	103.43	100.58	141.50	93.04
61	TRIOS 3	SLA	RSBFL	64.80	62.00	105.67	55.00
62	TRIOS 3	SLA	RSBFL	65.65	63.10	106.83	56.10
63	TRIOS 3	SLA	RSBFL	66.30	63.20	106.83	56.50
64	TRIOS 3	SLA	RSBFL	70.80	68.00	110.67	61.00
65	TRIOS 3	SLA	RSBFL	67.80	64.00	108.67	58.00
66	TRIOS 3	SLA	RSBFL	68.72	67.34	107.34	59.47
67	TRIOS 3	SLA	RSBFL	65.93	64.56	105.87	56.29
68	TRIOS 3	SLA	RSBFL	69.41	68.12	108.42	60.12
69	TRIOS 3	SLA	RSBFL	67.28	63.79	106.23	57.84
70	TRIOS 3	SLA	RSBFL	70.05	67.91	107.56	61.03
71	TRIOS 3	SLA	RSBFL	66.64	65.62	106.75	58.62
72	TRIOS 3	SLA	RSBFL	69.89	68.47	108.17	59.88
73	TRIOS 3	SLA	RSBFL	67.54	62.85	106.58	56.74
74	TRIOS 3	SLA	RSBFL	65.18	67.58	107.92	61.12
75	TRIOS 3	SLA	RSBFL	70.11	64.43	105.61	57.38
76	TRIOS 3	SLA	RSBFL	68.23	67.02	108.61	59.21
77	TRIOS 3	SLA	RSBFL	69.58	66.13	106.19	60.61
78	TRIOS 3	SLA	RSBFL	66.45	63.94	107.11	58.03
79	TRIOS 3	SLA	RSBFL	68.97	68.23	105.29	60.29
80	TRIOS 3	SLA	RSBFL	64.82	65.28	108.25	55.92
81	TRIOS 3	SLA	RSBFL	69.13	66.79	106.83	60.87
82	TRIOS 3	SLA	RSBFL	65.71	62.71	107.48	56.41
83	TRIOS 3	SLA	RSBFL	68.14	67.05	106.67	58.79
84	TRIOS 3	SLA	RSBFL	67.91	63.48	105.93	61.56
85	TRIOS 3	SLA	RSBFL	70.23	68.03	108.93	59.63
86	TRIOS 3	SLA	RSBFL	66.02	66.47	107.08	57.53
87	TRIOS 3	SLA	RSBFL	69.05	65.15	105.71	61.11
88	TRIOS 3	SLA	RSBFL	67.66	68.36	108.04	58.25
89	TRIOS 3	SLA	RSBFL	66.88	64.98	106.31	56.94
90	TRIOS 3	SLA	RSBFL	64.99	67.71	107.65	60.42
91	TRIOS 3	DLP	CFL	235.90	233.10	276.77	226.10
92	TRIOS 3	DLP	CFL	242.45	239.80	283.27	232.10
93	TRIOS 3	DLP	CFL	236.90	233.10	277.77	227.10
94	TRIOS 3	DLP	CFL	237.20	234.50	277.90	228.50
95	TRIOS 3	DLP	CFL	237.60	234.20	278.30	227.80
96	TRIOS 3	DLP	CFL	239.42	236.58	278.46	230.54
97	TRIOS 3	DLP	CFL	237.89	234.92	280.71	227.84
98	TRIOS 3	DLP	CFL	241.17	239.14	277.53	228.91
99	TRIOS 3	DLP	CFL	236.56	237.45	281.12	231.62
100	TRIOS 3	DLP	CFL	240.11	233.76	279.34	227.49
101	TRIOS 3	DLP	CFL	237.68	238.09	277.98	229.36
102	TRIOS 3	DLP	CFL	241.83	234.61	283.44	232.21
103	TRIOS 3	DLP	CFL	238.74	239.29	278.25	228.12
104	TRIOS 3	DLP	CFL	240.59	237.02	280.57	230.09
105	TRIOS 3	DLP	CFL	239.03	236.27	279.84	231.44
106	TRIOS 3	DLP	CFL	242.26	239.73	281.65	227.62
107	TRIOS 3	DLP	CFL	236.94	233.89	283.12	229.73
108	TRIOS 3	DLP	CFL	241.52	235.88	278.93	230.68
109	TRIOS 3	DLP	CFL	238.11	238.56	282.18	229.11
110	TRIOS 3	DLP	CFL	240.92	236.34	279.62	232.47
111	TRIOS 3	DLP	CFL	237.32	233.54	281.90	227.14
112	TRIOS 3	DLP	CFL	242.14	239.05	276.72	231.01
113	TRIOS 3	DLP	CFL	238.47	234.74	283.33	230.11
114	TRIOS 3	DLP	CFL	239.91	237.63	280.29	229.52
115	TRIOS 3	DLP	CFL	241.29	238.17	277.27	232.13
116	TRIOS 3	DLP	CFL	236.77	234.50	282.42	228.77
117	TRIOS 3	DLP	CFL	241.68	237.89	277.81	230.81
118	TRIOS 3	DLP	CFL	238.33	233.12	279.10	228.60
119	TRIOS 3	DLP	CFL	242.01	239.48	282.60	231.88
120	TRIOS 3	DLP	CFL	239.60	234.22	278.71	227.92
121	TRIOS 3	DLP	RSFL	100.60	97.80	141.47	90.80
122	TRIOS 3	DLP	RSFL	107.30	105.50	148.03	97.50
123	TRIOS 3	DLP	RSFL	101.60	98.80	142.47	91.80
124	TRIOS 3	DLP	RSFL	102.00	99.20	142.87	92.20
125	TRIOS 3	DLP	RSFL	102.30	98.80	143.17	92.50
126	TRIOS 3	DLP	RSFL	106.48	103.47	144.83	95.63
127	TRIOS 3	DLP	RSFL	104.73	98.92	146.25	91.77
128	TRIOS 3	DLP	RSFL	101.65	102.34	141.96	97.14
129	TRIOS 3	DLP	RSFL	106.21	99.56	148.12	94.58
130	TRIOS 3	DLP	RSFL	103.89	104.21	144.57	96.21
131	TRIOS 3	DLP	RSFL	105.77	100.11	146.72	90.43
132	TRIOS 3	DLP	RSFL	101.33	104.89	145.34	95.11
133	TRIOS 3	DLP	RSFL	104.56	101.45	147.81	92.82
134	TRIOS 3	DLP	RSFL	102.11	100.83	142.45	96.67
135	TRIOS 3	DLP	RSFL	107.23	104.07	146.09	91.36
136	TRIOS 3	DLP	RSFL	103.14	102.16	145.67	96.44
137	TRIOS 3	DLP	RSFL	106.62	100.39	143.21	93.27
138	TRIOS 3	DLP	RSFL	101.78	105.28	148.59	97.02
139	TRIOS 3	DLP	RSFL	104.08	99.14	142.79	92.13
140	TRIOS 3	DLP	RSFL	106.39	102.62	147.28	95.89
141	TRIOS 3	DLP	RSFL	102.93	100.74	144.11	94.74
142	TRIOS 3	DLP	RSFL	105.22	103.01	148.23	91.58
143	TRIOS 3	DLP	RSFL	101.49	101.92	146.48	96.90
144	TRIOS 3	DLP	RSFL	106.76	103.56	142.14	90.82
145	TRIOS 3	DLP	RSFL	104.91	98.29	145.22	94.29
146	TRIOS 3	DLP	RSFL	107.12	101.13	143.87	92.66
147	TRIOS 3	DLP	RSFL	103.42	105.04	147.03	95.38
148	TRIOS 3	DLP	RSFL	105.59	99.80	142.63	93.98
149	TRIOS 3	DLP	RSFL	102.34	101.67	148.14	91.24
150	TRIOS 3	DLP	RSFL	107.38	104.73	145.91	97.56
151	TRIOS 3	DLP	RSBFL	67.10	64.30	107.97	57.30
152	TRIOS 3	DLP	RSBFL	71.95	71.00	116.00	64.00
153	TRIOS 3	DLP	RSBFL	68.08	64.80	108.97	58.30
154	TRIOS 3	DLP	RSBFL	68.50	65.30	109.37	58.40
155	TRIOS 3	DLP	RSBFL	68.80	64.00	109.67	55.00
156	TRIOS 3	DLP	RSBFL	69.38	68.22	114.36	62.87
157	TRIOS 3	DLP	RSBFL	70.11	65.91	110.47	58.74
158	TRIOS 3	DLP	RSBFL	67.84	69.54	113.92	63.29
159	TRIOS 3	DLP	RSBFL	69.72	70.11	109.14	59.42
160	TRIOS 3	DLP	RSBFL	70.52	67.34	111.78	60.51
161	TRIOS 3	DLP	RSBFL	71.26	70.92	114.25	61.19
162	TRIOS 3	DLP	RSBFL	69.04	64.67	112.63	58.31
163	TRIOS 3	DLP	RSBFL	68.99	66.81	108.90	64.12
164	TRIOS 3	DLP	RSBFL	71.45	70.58	113.61	61.57
165	TRIOS 3	DLP	RSBFL	70.21	69.37	115.02	62.04
166	TRIOS 3	DLP	RSBFL	67.63	64.79	110.59	59.77
167	TRIOS 3	DLP	RSBFL	69.57	69.98	113.48	63.68
168	TRIOS 3	DLP	RSBFL	70.74	67.56	111.12	60.09
169	TRIOS 3	DLP	RSBFL	67.47	71.16	116.33	64.87
170	TRIOS 3	DLP	RSBFL	71.88	66.23	108.73	59.54
171	TRIOS 3	DLP	RSBFL	68.35	67.94	112.41	62.23
172	TRIOS 3	DLP	RSBFL	71.61	70.63	115.55	60.85
173	TRIOS 3	DLP	RSBFL	70.88	65.48	110.34	63.13
174	TRIOS 3	DLP	RSBFL	67.29	68.72	113.05	58.92
175	TRIOS 3	DLP	RSBFL	69.86	69.85	112.09	61.41
176	TRIOS 3	DLP	RSBFL	68.76	71.03	115.87	60.28
177	TRIOS 3	DLP	RSBFL	70.38	65.29	109.67	63.94
178	TRIOS 3	DLP	RSBFL	69.17	66.55	113.23	57.99
179	TRIOS 3	DLP	RSBFL	68.24	70.47	116.14	61.66
180	TRIOS 3	DLP	RSBFL	71.05	64.88	111.51	64.33
