# Supplementary material for: Advanced superimposition method to evaluate the marginal and internal fit of ceramic crowns fabricated using heat pressing techniques
Source: PeerJ. 2025 Apr 3;13:e19117. doi: 10.7717/peerj.19117 (PMC11972566; doi:10.7717/peerj.19117)
Supplement: Supplemental Information 2 [file peerj-13-19117-s002.pdf]

SUPPELEMENTARY TABLES

TABLE -S-1- Post Hoc Comparisons - IOS ✻ TECHNIQUE ✻ FINISHLINE at **MARGIN**

Post Hoc Comparisons - IOS ✻ TECHNIQUE ✻ FINISHLINE

**Comparison**											
**IOS**	**TECHNIQUE**	**FINISHLINE**		**IOS**	**TECHNIQUE**	**FINISHLINE**	**Mean Difference**	**SE**	**df**	**t**	**p_{tukey}**
MEDIT-i700	SLA	CFL	-	MEDIT-i700	SLA	RSFL	137.93	0.450	348	306.80	< .001
			-	MEDIT-i700	SLA	RSBFL	174.04	0.450	348	387.12	< .001
			-	MEDIT-i700	DLP	CFL	-6.19	0.450	348	-13.76	< .001
			-	MEDIT-i700	DLP	RSFL	132.70	0.450	348	295.15	< .001
			-	MEDIT-i700	DLP	RSBFL	169.41	0.450	348	376.82	< .001
			-	TRIOS3	SLA	CFL	-15.44	0.450	348	-34.34	< .001

Post Hoc Comparisons - IOS ✻ TECHNIQUE ✻ FINISHLINE

**Comparison**											
**IOS**	**TECHNIQUE**	**FINISHLINE**		**IOS**	**TECHNIQUE**	**FINISHLINE**	**Mean Difference**	**SE**	**df**	**t**	**p_{tukey}**
			-	TRIOS 3	SLA	RSFL	117.92	0.450	348	262.30	< .001
			-	TRIOS 3	SLA	RSBFL	151.02	0.450	348	335.91	< .001
			-	TRIOS 3	DLP	CFL	-20.66	0.450	348	-45.96	< .001
			-	TRIOS 3	DLP	RSFL	114.45	0.450	348	254.57	< .001
			-	TRIOS 3	DLP	RSBFL	149.16	0.450	348	331.76	< .001
		RSFL	-	MEDIT-i700	SLA	RSBFL	36.11	0.450	348	80.32	< .001
			-	MEDIT-i700	DLP	CFL	-144.12	0.450	348	-320.55	< .001
			-	MEDIT-i700	DLP	RSFL	-5.24	0.450	348	-11.65	< .001

Post Hoc Comparisons - IOS ✻ TECHNIQUE ✻ FINISHLINE

Comparison											
**IOS**	**TECHNIQUE**	**FINISHLINE**		**IOS**	**TECHNIQUE**	**FINISHLINE**	**Mean Difference**	**SE**	**df**	**t**	**p_{tukey}**
			-	MEDIT-i700	DLP	RSBFL	31.48	0.450	348	70.02	< .001
			-	TRIOS3	SLA	CFL	-153.37	0.450	348	-341.14	< .001
			-	TRIOS3	SLA	RSFL	-20.01	0.450	348	-44.50	< .001
			-	TRIOS3	SLA	RSBFL	13.09	0.450	348	29.11	< .001
			-	TRIOS3	DLP	CFL	-158.59	0.450	348	-352.75	< .001
			-	TRIOS3	DLP	RSFL	-23.48	0.450	348	-52.23	< .001
			-	TRIOS3	DLP	RSBFL	11.22	0.450	348	24.96	< .001
		RSBFL	-	MEDIT-i700	DLP	CFL	-180.23	0.450	348	-400.87	< .001

Post Hoc Comparisons - IOS ✻ TECHNIQUE ✻ FINISHLINE

Comparison											
**IOS**	**TECHNIQUE**	**FINISHLINE**		**IOS**	**TECHNIQUE**	**FINISHLINE**	**Mean Difference**	**SE**	**df**	**t**	**p_{tukey}**
			-	MEDIT-i700	DLP	RSFL	-41.35	0.450	348	-91.97	< .001
			-	MEDIT-i700	DLP	RSBFL	-4.63	0.450	348	-10.30	< .001
			-	TRIOS3	SLA	CFL	-189.48	0.450	348	-421.45	< .001
			-	TRIOS3	SLA	RSFL	-56.12	0.450	348	-124.82	< .001
			-	TRIOS3	SLA	RSBFL	-23.02	0.450	348	-51.21	< .001
			-	TRIOS3	DLP	CFL	-194.70	0.450	348	-433.07	< .001
			-	TRIOS3	DLP	RSFL	-59.59	0.450	348	-132.54	< .001
			-	TRIOS3	DLP	RSBFL	-24.89	0.450	348	-55.35	< .001

Post Hoc Comparisons - IOS ✻ TECHNIQUE ✻ FINISHLINE

Comparison											
**IOS**	**TECHNIQUE**	**FINISHLINE**		**IOS**	**TECHNIQUE**	**FINISHLINE**	**Mean Difference**	**SE**	**df**	**t**	**p_{tukey}**
	DLP	CFL	-	MEDIT-i700	DLP	RSFL	138.88	0.450	348	308.91	< .001
			-	MEDIT-i700	DLP	RSBFL	175.60	0.450	348	390.57	< .001
			-	TRIOS3	SLA	CFL	-9.25	0.450	348	-20.58	< .001
			-	TRIOS3	SLA	RSFL	124.11	0.450	348	276.05	< .001
			-	TRIOS3	SLA	RSBFL	157.21	0.450	348	349.67	< .001
			-	TRIOS3	DLP	CFL	-14.48	0.450	348	-32.20	< .001
			-	TRIOS3	DLP	RSFL	120.64	0.450	348	268.33	< .001
			-	TRIOS3	DLP	RSBFL	155.34	0.450	348	345.52	< .001

Post Hoc Comparisons - IOS ✻ TECHNIQUE ✻ FINISHLINE

**Comparison**											
**IOS**	**TECHNIQUE**	**FINISHLINE**		**IOS**	**TECHNIQUE**	**FINISHLINE**	**Mean Difference**	**SE**	**df**	**t**	**p_{tukey}**
		RSFL	-	MEDIT-i700	DLP	RSBFL	36.72	0.450	348	81.67	< .001
			-	TRIOS3	SLA	CFL	-148.13	0.450	348	-329.49	< .001
			-	TRIOS3	SLA	RSFL	-14.77	0.450	348	-32.85	< .001
			-	TRIOS3	SLA	RSBFL	18.33	0.450	348	40.76	< .001
			-	TRIOS3	DLP	CFL	-153.36	0.450	348	-341.10	< .001
			-	TRIOS3	DLP	RSFL	-18.24	0.450	348	-40.58	< .001
			-	TRIOS3	DLP	RSBFL	16.46	0.450	348	36.61	< .001
		RSBFL	-	TRIOS3	SLA	CFL	-184.85	0.450	348	-411.15	< .001
			-	TRIOS3	SLA	RSFL	-51.49	0.450	348	-114.52	< .001

Post Hoc Comparisons - IOS ✻ TECHNIQUE ✻ FINISHLINE

**Comparison**											
**IOS**	**TECHNIQUE**	**FINISHLINE**		**IOS**	**TECHNIQUE**	**FINISHLINE**	**Mean Difference**	**SE**	**df**	**t**	**p_{tukey}**
			-	TRIOS 3	SLA	RSBFL	-18.39	0.450	348	-40.91	< .001
			-	TRIOS 3	DLP	CFL	-190.07	0.450	348	-422.77	< .001
			-	TRIOS 3	DLP	RSFL	-54.96	0.450	348	-122.24	< .001
			-	TRIOS 3	DLP	RSBFL	-20.26	0.450	348	-45.05	< .001
TRIOS 3	SLA	CFL	-	TRIOS 3	SLA	RSFL	133.36	0.450	348	296.63	< .001
			-	TRIOS 3	SLA	RSBFL	166.46	0.450	348	370.25	< .001
			-	TRIOS 3	DLP	CFL	-5.22	0.450	348	-11.62	< .001
			-	TRIOS 3	DLP	RSFL	129.89	0.450	348	288.91	< .001
			-	TRIOS 3	DLP	RSBFL	164.59	0.450	348	366.10	< .001
		RSFL	-	TRIOS 3	SLA	RSBFL	33.10	0.450	348	73.61	< .001

Post Hoc Comparisons - IOS ✻ TECHNIQUE ✻ FINISHLINE

Comparison											
**IOS**	**TECHNIQUE**	**FINISHLINE**		**IOS**	**TECHNIQUE**	**FINISHLINE**	**Mean Difference**	**SE**	**df**	**t**	**p_{tukey}**
			-	TRIOS 3	DLP	CFL	-138.59	0.450	348	-308.25	< .001
			-	TRIOS 3	DLP	RSFL	-3.47	0.450	348	-7.72	< .001
			-	TRIOS 3	DLP	RSBFL	31.23	0.450	348	69.47	< .001
		RSBFL	-	TRIOS 3	DLP	CFL	-171.68	0.450	348	-381.87	< .001
			-	TRIOS 3	DLP	RSFL	-36.57	0.450	348	-81.34	< .001
			-	TRIOS 3	DLP	RSBFL	-1.87	0.450	348	-4.15	0.002
	DLP	CFL	-	TRIOS 3	DLP	RSFL	135.11	0.450	348	300.53	< .001
			-	TRIOS 3	DLP	RSBFL	169.82	0.450	348	377.72	< .001
		RSFL	-	TRIOS 3	DLP	RSBFL	34.70	0.450	348	77.19	< .001

Note. Comparisons are based on estimated marginal means

TABLE -S-2- Post Hoc Comparisons - IOS ✻ TECHNIQUE ✻ FINISHLINE at **B-AXIAL**

Post Hoc Comparisons - IOS ✻ TECHNIQUE ✻ FINISHLINE

**Comparison**											
**IOS**	**TECHNIQUE**	**FINISHLINE**		**IOS**	**TECHNIQUE**	**FINISHLINE**	**Mean Difference**	**SE**	**df**	**t**	p_{tukey}
MEDIT-i700	SLA	CFL	-	MEDIT-i700	SLA	RSFL	138.38	0.499	348	277.54	< .001
			-	MEDIT-i700	SLA	RSBFL	174.82	0.499	348	350.62	< .001
			-	MEDIT-i700	DLP	CFL	-5.17	0.499	348	-10.37	< .001
			-	MEDIT-i700	DLP	RSFL	133.31	0.499	348	267.37	< .001
			-	MEDIT-i700	DLP	RSBFL	169.93	0.499	348	340.81	< .001
			-	TRIOS3	SLA	CFL	-16.02	0.499	348	-32.12	< .001
			-	TRIOS3	SLA	RSFL	118.23	0.499	348	237.13	< .001

Post Hoc Comparisons - IOS ✻ TECHNIQUE ✻ FINISHLINE

Comparison											
IOS	TECHNIQUE	FINISHLINE		IOS	TECHNIQUE	FINISHLINE	Mean Difference	SE	df	t	p_{tukey}
			-	TRIOS 3	SLA	RSBFL	151.09	0.499	348	303.02	< .001
			-	TRIOS 3	DLP	CFL	-19.46	0.499	348	-39.02	< .001
			-	TRIOS 3	DLP	RSFL	115.18	0.499	348	231.01	< .001
			-	TRIOS 3	DLP	RSBFL	149.05	0.499	348	298.93	< .001
		RSFL	-	MEDIT-i700	SLA	RSBFL	36.44	0.499	348	73.08	< .001
			-	MEDIT-i700	DLP	CFL	-143.56	0.499	348	-287.92	< .001
			-	MEDIT-i700	DLP	RSFL	-5.07	0.499	348	-10.17	< .001
			-	MEDIT-i700	DLP	RSBFL	31.55	0.499	348	63.27	< .001

Post Hoc Comparisons - IOS ✻ TECHNIQUE ✻ FINISHLINE

**Comparison**											
**IOS**	**TECHNIQUE**	**FINISHLINE**		**IOS**	**TECHNIQUE**	**FINISHLINE**	**Mean Difference**	**SE**	**df**	**t**	**p_{tukey}**
			-	TRIOS 3	SLA	CFL	-154.40	0.499	348	-309.66	< .001
			-	TRIOS 3	SLA	RSFL	-20.15	0.499	348	-40.41	< .001
			-	TRIOS 3	SLA	RSBFL	12.70	0.499	348	25.47	< .001
			-	TRIOS 3	DLP	CFL	-157.84	0.499	348	-316.56	< .001
			-	TRIOS 3	DLP	RSFL	-23.20	0.499	348	-46.54	< .001
			-	TRIOS 3	DLP	RSBFL	10.66	0.499	348	21.39	< .001
		RSBFL	-	MEDIT-i700	DLP	CFL	-179.99	0.499	348	-360.99	< .001
			-	MEDIT-i700	DLP	RSFL	-41.51	0.499	348	-83.24	< .001

Post Hoc Comparisons - IOS ✻ TECHNIQUE ✻ FINISHLINE

**Comparison**											
**IOS**	**TECHNIQUE**	**FINISHLINE**		**IOS**	**TECHNIQUE**	**FINISHLINE**	**Mean Difference**	**SE**	**df**	**t**	**p_{tukey}**
			-	MEDIT-i700	DLP	RSBFL	-4.89	0.499	348	-9.81	< .001
			-	TRIOS3	SLA	CFL	-190.84	0.499	348	-382.74	< .001
			-	TRIOS3	SLA	RSFL	-56.59	0.499	348	-113.49	< .001
			-	TRIOS3	SLA	RSBFL	-23.73	0.499	348	-47.60	< .001
			-	TRIOS3	DLP	CFL	-194.28	0.499	348	-389.64	< .001
			-	TRIOS3	DLP	RSFL	-59.64	0.499	348	-119.61	< .001
			-	TRIOS3	DLP	RSBFL	-25.77	0.499	348	-51.69	< .001
	DLP	CFL	-	MEDIT-i700	DLP	RSFL	138.49	0.499	348	277.75	< .001

Post Hoc Comparisons - IOS ✻ TECHNIQUE ✻ FINISHLINE

Comparison											
**IOS**	**TECHNIQUE**	**FINISHLINE**		**IOS**	**TECHNIQUE**	**FINISHLINE**	**Mean Difference**	**SE**	**df**	**t**	**p_{tukey}**
			-	MEDIT-i700	DLP	RSBFL	175.10	0.499	348	351.18	< .001
			-	TRIOS3	SLA	CFL	-10.84	0.499	348	-21.75	< .001
			-	TRIOS3	SLA	RSFL	123.41	0.499	348	247.50	< .001
			-	TRIOS3	SLA	RSBFL	156.26	0.499	348	313.39	< .001
			-	TRIOS3	DLP	CFL	-14.28	0.499	348	-28.65	< .001
			-	TRIOS3	DLP	RSFL	120.35	0.499	348	241.38	< .001
			-	TRIOS3	DLP	RSBFL	154.22	0.499	348	309.30	< .001
		RSFL	-	MEDIT-i700	DLP	RSBFL	36.62	0.499	348	73.44	< .001
			-	TRIOS3	SLA	CFL	-149.33	0.499	348	-299.50	< .001

Post Hoc Comparisons - IOS ✻ TECHNIQUE ✻ FINISHLINE

**Comparison**											
**IOS**	**TECHNIQUE**	**FINISHLINE**		**IOS**	**TECHNIQUE**	**FINISHLINE**	**Mean Difference**	**SE**	**df**	**t**	p_{tukey}
			-	TRIOS 3	SLA	RSFL	-15.08	0.499	348	-30.24	< .001
			-	TRIOS 3	SLA	RSBFL	17.77	0.499	348	35.64	< .001
			-	TRIOS 3	DLP	CFL	-152.77	0.499	348	-306.40	< .001
			-	TRIOS 3	DLP	RSFL	-18.13	0.499	348	-36.37	< .001
			-	TRIOS 3	DLP	RSBFL	15.73	0.499	348	31.56	< .001
		RSBFL	-	TRIOS 3	SLA	CFL	-185.95	0.499	348	-372.93	< .001
			-	TRIOS 3	SLA	RSFL	-51.70	0.499	348	-103.68	< .001
			-	TRIOS 3	SLA	RSBFL	-18.84	0.499	348	-37.79	< .001
			-	TRIOS 3	DLP	CFL	-189.39	0.499	348	-379.83	< .001
			-	TRIOS 3	DLP	RSFL	-54.75	0.499	348	-109.80	< .001

Post Hoc Comparisons - IOS ✻ TECHNIQUE ✻ FINISHLINE

**Comparison**											
**IOS**	**TECHNIQUE**	**FINISHLINE**		**IOS**	**TECHNIQUE**	**FINISHLINE**	**Mean Difference**	**SE**	**df**	**t**	**p_{tukey}**
			-	TRIOS 3	DLP	RSBFL	-20.88	0.499	348	-41.88	< .001
TRIOS 3	SLA	CFL	-	TRIOS 3	SLA	RSFL	134.25	0.499	348	269.25	< .001
			-	TRIOS 3	SLA	RSBFL	167.10	0.499	348	335.14	< .001
			-	TRIOS 3	DLP	CFL	-3.44	0.499	348	-6.90	< .001
			-	TRIOS 3	DLP	RSFL	131.20	0.499	348	263.13	< .001
			-	TRIOS 3	DLP	RSBFL	165.06	0.499	348	331.05	< .001
		RSFL	-	TRIOS 3	SLA	RSBFL	32.85	0.499	348	65.89	< .001
			-	TRIOS 3	DLP	CFL	-137.69	0.499	348	-276.15	< .001
			-	TRIOS 3	DLP	RSFL	-3.05	0.499	348	-6.12	< .001
			-	TRIOS 3	DLP	RSBFL	30.81	0.499	348	61.80	< .001

Post Hoc Comparisons - IOS ✻ TECHNIQUE ✻ FINISHLINE

Comparison											
**IOS**	**TECHNIQUE**	**FINISHLINE**		**IOS**	**TECHNIQUE**	**FINISHLINE**	**Mean Difference**	**SE**	**df**	**t**	**p_{tukey}**
		RSBFL	-	TRIOS3	DLP	CFL	-170.54	0.499	348	-342.04	< .001
			-	TRIOS3	DLP	RSFL	-35.90	0.499	348	-72.01	< .001
			-	TRIOS3	DLP	RSBFL	-2.04	0.499	348	-4.09	0.003
	DLP	CFL	-	TRIOS3	DLP	RSFL	134.64	0.499	348	270.03	< .001
			-	TRIOS3	DLP	RSBFL	168.50	0.499	348	337.95	< .001
		RSFL	-	TRIOS3	DLP	RSBFL	33.87	0.499	348	67.92	< .001

Note. Comparisons are based on estimated marginal means

TABLE -S-3- Post Hoc Comparisons - IOS ✻ TECHNIQUE ✻ FINISHLINE at **OCCLUSAL**

Post Hoc Comparisons - IOS ✻ TECHNIQUE ✻ FINISHLINE

**Comparison**											
**IOS**	**TECHNIQUE**	**FINISHLINE**		**IOS**	**TECHNIQUE**	**FINISHLINE**	**Mean Difference**	**SE**	**df**	**t**	**p_{tukey}**
MEDIT - i700	SLA	CFL	-	MEDIT - i700	SLA	RSFL	138.22	0.464	348	297.91	< .001
			-	MEDIT - i700	SLA	RSBFL	174.17	0.464	348	375.40	< .001
			-	MEDIT - i700	DLP	CFL	-6.59	0.464	348	-14.21	< .001
			-	MEDIT - i700	DLP	RSFL	132.26	0.464	348	285.06	< .001
			-	MEDIT - i700	DLP	RSBFL	168.03	0.464	348	362.17	< .001
			-	TRIOS 3	SLA	CFL	-15.90	0.464	348	-34.27	< .001
			-	TRIOS 3	SLA	RSFL	117.59	0.464	348	253.45	< .001

Post Hoc Comparisons - IOS ✻ TECHNIQUE ✻ FINISHLINE

Comparison							Mean Difference	SE	df	t	p_{tukey}
**IOS**	**TECHNIQUE**	**FINISHLINE**		**IOS**	**TECHNIQUE**	**FINISHLINE**					
			-	TRIOS3	SLA	RSBFL	152.09	0.464	348	327.81	< .001
			-	TRIOS3	DLP	CFL	-20.63	0.464	348	-44.46	< .001
			-	TRIOS3	DLP	RSFL	114.15	0.464	348	246.03	< .001
			-	TRIOS3	DLP	RSBFL	147.06	0.464	348	316.96	< .001
		RSFL	-	MEDIT-i700	SLA	RSBFL	35.95	0.464	348	77.49	< .001
			-	MEDIT-i700	DLP	CFL	-144.81	0.464	348	-312.12	< .001
			-	MEDIT-i700	DLP	RSFL	-5.96	0.464	348	-12.85	< .001
			-	MEDIT-i700	DLP	RSBFL	29.81	0.464	348	64.26	< .001

Post Hoc Comparisons - IOS ✻ TECHNIQUE ✻ FINISHLINE

Comparison											
**IOS**	**TECHNIQUE**	**FINISHLINE**		**IOS**	**TECHNIQUE**	**FINISHLINE**	**Mean Difference**	**SE**	**df**	**t**	**p_{tukey}**
			-	TRIOS 3	SLA	CFL	-154.12	0.464	348	-332.18	< .001
			-	TRIOS 3	SLA	RSFL	-20.63	0.464	348	-44.47	< .001
			-	TRIOS 3	SLA	RSBFL	13.87	0.464	348	29.90	< .001
			-	TRIOS 3	DLP	CFL	-158.85	0.464	348	-342.37	< .001
			-	TRIOS 3	DLP	RSFL	-24.07	0.464	348	-51.88	< .001
			-	TRIOS 3	DLP	RSBFL	8.84	0.464	348	19.05	< .001
		RSBFL	-	MEDIT-i700	DLP	CFL	-180.76	0.464	348	-389.60	< .001
			-	MEDIT-i700	DLP	RSFL	-41.91	0.464	348	-90.34	< .001

Post Hoc Comparisons - IOS ✻ TECHNIQUE ✻ FINISHLINE

Comparison											
**IOS**	**TECHNIQUE**	**FINISHLINE**		**IOS**	**TECHNIQUE**	**FINISHLINE**	**Mean Difference**	**SE**	**df**	**t**	**p_{tukey}**
			-	MEDIT-i700	DLP	RSBFL	-6.14	0.464	348	-13.23	< .001
			-	TRIOS3	SLA	CFL	-190.07	0.464	348	-409.67	< .001
			-	TRIOS3	SLA	RSFL	-56.58	0.464	348	-121.95	< .001
			-	TRIOS3	SLA	RSBFL	-22.08	0.464	348	-47.59	< .001
			-	TRIOS3	DLP	CFL	-194.80	0.464	348	-419.86	< .001
			-	TRIOS3	DLP	RSFL	-60.02	0.464	348	-129.36	< .001
			-	TRIOS3	DLP	RSBFL	-27.11	0.464	348	-58.43	< .001
	DLP	CFL	-	MEDIT-i700	DLP	RSFL	138.85	0.464	348	299.27	< .001

Post Hoc Comparisons - IOS ✻ TECHNIQUE ✻ FINISHLINE

**Comparison**											
**IOS**	**TECHNIQUE**	**FINISHLINE**		**IOS**	**TECHNIQUE**	**FINISHLINE**	**Mean Difference**	**SE**	**df**	**t**	**p_{tukey}**
			-	MEDIT-i700	DLP	RSBFL	174.63	0.464	348	376.38	< .001
			-	TRIOS3	SLA	CFL	-9.31	0.464	348	-20.06	< .001
			-	TRIOS3	SLA	RSFL	124.18	0.464	348	267.65	< .001
			-	TRIOS3	SLA	RSBFL	158.69	0.464	348	342.02	< .001
			-	TRIOS3	DLP	CFL	-14.04	0.464	348	-30.26	< .001
			-	TRIOS3	DLP	RSFL	120.74	0.464	348	260.24	< .001
			-	TRIOS3	DLP	RSBFL	153.65	0.464	348	331.17	< .001
		RSFL	-	MEDIT-i700	DLP	RSBFL	35.78	0.464	348	77.11	< .001
			-	TRIOS3	SLA	CFL	-148.16	0.464	348	-319.33	< .001

Post Hoc Comparisons - IOS ✻ TECHNIQUE ✻ FINISHLINE

Comparison											
IOS	TECHNIQUE	FINISHLINE		IOS	TECHNIQUE	FINISHLINE	Mean Difference	SE	df	t	p_{tukey}
			-	TRIOS3	SLA	RSFL	-14.67	0.464	348	-31.61	< .001
			-	TRIOS3	SLA	RSBFL	19.84	0.464	348	42.75	< .001
			-	TRIOS3	DLP	CFL	-152.89	0.464	348	-329.52	< .001
			-	TRIOS3	DLP	RSFL	-18.11	0.464	348	-39.02	< .001
			-	TRIOS3	DLP	RSBFL	14.80	0.464	348	31.91	< .001
		RSBFL	-	TRIOS3	SLA	CFL	-183.94	0.464	348	-396.44	< .001
			-	TRIOS3	SLA	RSFL	-50.44	0.464	348	-108.72	< .001
			-	TRIOS3	SLA	RSBFL	-15.94	0.464	348	-34.36	< .001
			-	TRIOS3	DLP	CFL	-188.66	0.464	348	-406.63	< .001
			-	TRIOS3	DLP	RSFL	-53.88	0.464	348	-116.13	< .001

Post Hoc Comparisons - IOS ✻ TECHNIQUE ✻ FINISHLINE

Comparison											
IOS	TECHNIQUE	FINISHLINE		IOS	TECHNIQUE	FINISHLINE	Mean Difference	SE	df	t	p_{tukey}
			-	TRIOS 3	DLP	RSBFL	-20.97	0.464	348	-45.20	< .001
TRIOS 3	SLA	CFL	-	TRIOS 3	SLA	RSFL	133.49	0.464	348	287.72	< .001
			-	TRIOS 3	SLA	RSBFL	167.99	0.464	348	362.08	< .001
			-	TRIOS 3	DLP	CFL	-4.73	0.464	348	-10.19	< .001
			-	TRIOS 3	DLP	RSFL	130.05	0.464	348	280.30	< .001
			-	TRIOS 3	DLP	RSBFL	162.96	0.464	348	351.23	< .001
		RSFL	-	TRIOS 3	SLA	RSBFL	34.50	0.464	348	74.36	< .001
			-	TRIOS 3	DLP	CFL	-138.22	0.464	348	-297.91	< .001
			-	TRIOS 3	DLP	RSFL	-3.44	0.464	348	-7.41	< .001
			-	TRIOS 3	DLP	RSBFL	29.47	0.464	348	63.52	< .001

Post Hoc Comparisons - IOS ✻ TECHNIQUE ✻ FINISHLINE

**Comparison**											
**IOS**	**TECHNIQUE**	**FINISHLINE**		**IOS**	**TECHNIQUE**	**FINISHLINE**	**Mean Difference**	**SE**	**df**	**t**	p_{tukey}
		RSBFL	-	TRIOS3	DLP	CFL	-172.72	0.464	348	-372.27	< .001
			-	TRIOS3	DLP	RSFL	-37.94	0.464	348	-81.78	< .001
			-	TRIOS3	DLP	RSBFL	-5.03	0.464	348	-10.85	< .001
	DLP	CFL	-	TRIOS3	DLP	RSFL	134.78	0.464	348	290.50	< .001
			-	TRIOS3	DLP	RSBFL	167.69	0.464	348	361.43	< .001
		RSFL	-	TRIOS3	DLP	RSBFL	32.91	0.464	348	70.93	< .001

Note. Comparisons are based on estimated marginal means

TABLE -S-4- Post Hoc Comparisons - IOS ✻ TECHNIQUE ✻ FINISHLINE at **P-AXIAL**

Post Hoc Comparisons - IOS ✻ TECHNIQUE ✻ FINISHLINE

**Comparison**											
**IOS**	**TECHNIQUE**	**FINISHLINE**		**IOS**	**TECHNIQUE**	**FINISHLINE**	**Mean Difference**	**SE**	**df**	**t**	p_{tukey}
MEDIT - i700	SLA	CFL	-	MEDIT - i700	SLA	RSFL	134.32	0.527	348	254.95	< .001
			-	MEDIT - i700	SLA	RSBFL	173.90	0.527	348	330.07	< .001
			-	MEDIT - i700	DLP	CFL	-5.64	0.527	348	-10.70	< .001
			-	MEDIT - i700	DLP	RSFL	132.11	0.527	348	250.76	< .001
			-	MEDIT - i700	DLP	RSBFL	168.49	0.527	348	319.80	< .001
			-	TRIOS 3	SLA	CFL	-16.74	0.527	348	-31.76	< .001
			-	TRIOS 3	SLA	RSFL	117.03	0.527	348	222.14	< .001

Post Hoc Comparisons - IOS ✻ TECHNIQUE ✻ FINISHLINE

**Comparison**											
**IOS**	**TECHNIQUE**	**FINISHLINE**		**IOS**	**TECHNIQUE**	**FINISHLINE**	**Mean Difference**	**SE**	**df**	**t**	**p_{tukey}**
			-	TRIOS 3	SLA	RSBFL	150.20	0.527	348	285.08	< .001
			-	TRIOS 3	DLP	CFL	-20.67	0.527	348	-39.24	< .001
			-	TRIOS 3	DLP	RSFL	114.87	0.527	348	218.03	< .001
			-	TRIOS 3	DLP	RSBFL	147.96	0.527	348	280.84	< .001
		RSFL	-	MEDIT-i700	SLA	RSBFL	39.58	0.527	348	75.13	< .001
			-	MEDIT-i700	DLP	CFL	-139.96	0.527	348	-265.65	< .001
			-	MEDIT-i700	DLP	RSFL	-2.21	0.527	348	-4.19	0.002
			-	MEDIT-i700	DLP	RSBFL	34.17	0.527	348	64.85	< .001

Post Hoc Comparisons - IOS ✻ TECHNIQUE ✻ FINISHLINE

Comparison											
**IOS**	**TECHNIQUE**	**FINISHLINE**		**IOS**	**TECHNIQUE**	**FINISHLINE**	**Mean Difference**	**SE**	**df**	**t**	**p_{tukey}**
			-	TRIOS 3	SLA	CFL	-151.05	0.527	348	-286.71	< .001
			-	TRIOS 3	SLA	RSFL	-17.28	0.527	348	-32.80	< .001
			-	TRIOS 3	SLA	RSBFL	15.88	0.527	348	30.14	< .001
			-	TRIOS 3	DLP	CFL	-154.99	0.527	348	-294.19	< .001
			-	TRIOS 3	DLP	RSFL	-19.45	0.527	348	-36.91	< .001
			-	TRIOS 3	DLP	RSBFL	13.64	0.527	348	25.89	< .001
		RSBFL	-	MEDIT-i700	DLP	CFL	-179.54	0.527	348	-340.78	< .001
			-	MEDIT-i700	DLP	RSFL	-41.79	0.527	348	-79.32	< .001

Post Hoc Comparisons - IOS ✻ TECHNIQUE ✻ FINISHLINE

**Comparison**											
**IOS**	**TECHNIQUE**	**FINISHLINE**		**IOS**	**TECHNIQUE**	**FINISHLINE**	**Mean Difference**	**SE**	**df**	**t**	**p_{tukey}**
			-	MEDIT-i700	DLP	RSBFL	-5.41	0.527	348	-10.27	< .001
			-	TRIOS3	SLA	CFL	-190.63	0.527	348	-361.84	< .001
			-	TRIOS3	SLA	RSFL	-56.86	0.527	348	-107.93	< .001
			-	TRIOS3	SLA	RSBFL	-23.70	0.527	348	-44.99	< .001
			-	TRIOS3	DLP	CFL	-194.57	0.527	348	-369.31	< .001
			-	TRIOS3	DLP	RSFL	-59.03	0.527	348	-112.04	< .001
			-	TRIOS3	DLP	RSBFL	-25.94	0.527	348	-49.24	< .001
	DLP	CFL	-	MEDIT-i700	DLP	RSFL	137.75	0.527	348	261.46	< .001

Post Hoc Comparisons - IOS ✻ TECHNIQUE ✻ FINISHLINE

**Comparison**											
**IOS**	**TECHNIQUE**	**FINISHLINE**		**IOS**	**TECHNIQUE**	**FINISHLINE**	**Mean Difference**	**SE**	**df**	**t**	**p_{tukey}**
			-	MEDIT-i700	DLP	RSBFL	174.12	0.527	348	330.50	< .001
			-	TRIOS3	SLA	CFL	-11.10	0.527	348	-21.06	< .001
			-	TRIOS3	SLA	RSFL	122.67	0.527	348	232.84	< .001
			-	TRIOS3	SLA	RSBFL	155.83	0.527	348	295.78	< .001
			-	TRIOS3	DLP	CFL	-15.04	0.527	348	-28.54	< .001
			-	TRIOS3	DLP	RSFL	120.51	0.527	348	228.73	< .001
			-	TRIOS3	DLP	RSBFL	153.60	0.527	348	291.54	< .001
		RSFL	-	MEDIT-i700	DLP	RSBFL	36.37	0.527	348	69.04	< .001
			-	TRIOS3	SLA	CFL	-148.85	0.527	348	-282.52	< .001

Post Hoc Comparisons - IOS ✻ TECHNIQUE ✻ FINISHLINE

Comparison											
**IOS**	**TECHNIQUE**	**FINISHLINE**		**IOS**	**TECHNIQUE**	**FINISHLINE**	**Mean Difference**	**SE**	**df**	**t**	**p_{tukey}**
			-	TRIOS 3	SLA	RSFL	-15.08	0.527	348	-28.62	< .001
			-	TRIOS 3	SLA	RSBFL	18.08	0.527	348	34.32	< .001
			-	TRIOS 3	DLP	CFL	-152.78	0.527	348	-290.00	< .001
			-	TRIOS 3	DLP	RSFL	-17.24	0.527	348	-32.73	< .001
			-	TRIOS 3	DLP	RSBFL	15.85	0.527	348	30.08	< .001
		RSBFL	-	TRIOS 3	SLA	CFL	-185.22	0.527	348	-351.56	< .001
			-	TRIOS 3	SLA	RSFL	-51.45	0.527	348	-97.66	< .001
			-	TRIOS 3	SLA	RSBFL	-18.29	0.527	348	-34.72	< .001
			-	TRIOS 3	DLP	CFL	-189.16	0.527	348	-359.04	< .001
			-	TRIOS 3	DLP	RSFL	-53.62	0.527	348	-101.77	< .001

Post Hoc Comparisons - IOS ✻ TECHNIQUE ✻ FINISHLINE

Comparison											
**IOS**	**TECHNIQUE**	**FINISHLINE**		**IOS**	**TECHNIQUE**	**FINISHLINE**	**Mean Difference**	**SE**	**df**	**t**	**p_{tukey}**
			-	TRIOS 3	DLP	RSBFL	-20.53	0.527	348	-38.96	< .001
TRIOS 3	SLA	CFL	-	TRIOS 3	SLA	RSFL	133.77	0.527	348	253.91	< .001
			-	TRIOS 3	SLA	RSBFL	166.93	0.527	348	316.85	< .001
			-	TRIOS 3	DLP	CFL	-3.94	0.527	348	-7.48	< .001
			-	TRIOS 3	DLP	RSFL	131.60	0.527	348	249.80	< .001
			-	TRIOS 3	DLP	RSBFL	164.69	0.527	348	312.60	< .001
		RSFL	-	TRIOS 3	SLA	RSBFL	33.16	0.527	348	62.94	< .001
			-	TRIOS 3	DLP	CFL	-137.71	0.527	348	-261.38	< .001
			-	TRIOS 3	DLP	RSFL	-2.16	0.527	348	-4.11	0.003
			-	TRIOS 3	DLP	RSBFL	30.92	0.527	348	58.70	< .001

Post Hoc Comparisons - IOS ✻ TECHNIQUE ✻ FINISHLINE

Comparison											
**IOS**	**TECHNIQUE**	**FINISHLINE**		**IOS**	**TECHNIQUE**	**FINISHLINE**	**Mean Difference**	**SE**	**df**	**t**	**p_{tukey}**
		RSBFL	-	TRIOS 3	DLP	CFL	-170.87	0.527	348	-324.32	< .001
			-	TRIOS 3	DLP	RSFL	-35.33	0.527	348	-67.05	< .001
			-	TRIOS 3	DLP	RSBFL	-2.24	0.527	348	-4.24	0.002
	DLP	CFL	-	TRIOS 3	DLP	RSFL	135.54	0.527	348	257.27	< .001
			-	TRIOS 3	DLP	RSBFL	168.63	0.527	348	320.08	< .001
		RSFL	-	TRIOS 3	DLP	RSBFL	33.09	0.527	348	62.81	< .001

Note. Comparisons are based on estimated marginal means
